# How Segmental Dynamics and Mesh Confinement Determine the Selective Diffusivity of Molecules in Cross-Linked Dense Polymer Networks

**DOI:** 10.1021/acscentsci.2c01373

**Published:** 2023-02-24

**Authors:** Baicheng Mei, Tsai-Wei Lin, Grant S. Sheridan, Christopher M. Evans, Charles E. Sing, Kenneth S. Schweizer

**Affiliations:** † Department of Materials Science, University of Illinois, Urbana, Illinois 61801, United States; ‡ Department of Chemistry, University of Illinois, Urbana, Illinois 61801, United States; § Department of Chemical & Biomolecular Engineering, University of Illinois, Urbana, Illinois 61801, United States; ∥ Materials Research Laboratory, University of Illinois, Urbana, Illinois 61801, United States

## Abstract

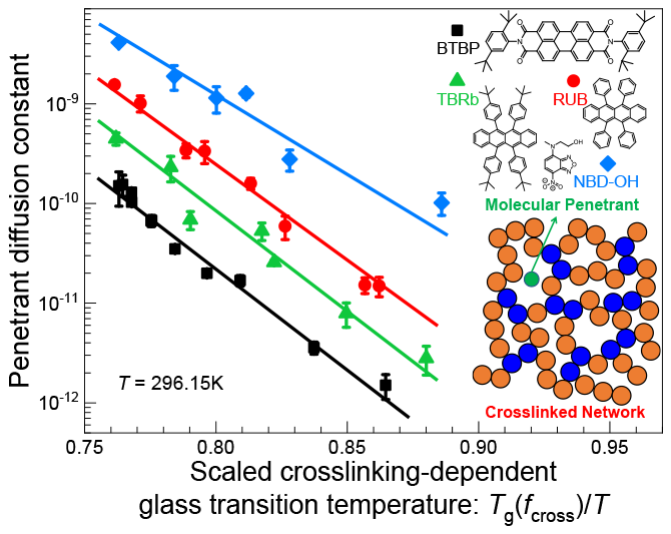

The diffusion of molecules (“penetrants”) of variable size, shape, and chemistry through dense cross-linked polymer networks is a fundamental scientific problem broadly relevant in materials, polymer, physical, and biological chemistry. Relevant applications include separation membranes, barrier materials, drug delivery, and nanofiltration. A major open question is the relationship between transport, thermodynamic state, and penetrant and polymer chemical structure. Here we combine experiment, simulation, and theory to unravel these competing effects on penetrant transport in rubbery and supercooled polymer permanent networks over a wide range of cross-link densities, size ratios, and temperatures. The crucial importance of the coupling of local penetrant hopping to polymer structural relaxation and the secondary importance of mesh confinement effects are established. Network cross-links strongly slow down nm-scale polymer relaxation, which greatly retards the activated penetrant diffusion. The demonstrated good agreement between experiment, simulation, and theory provides strong support for the size ratio (penetrant diameter to the polymer Kuhn length) as a key variable and the usefulness of coarse-grained simulation and theoretical models that average over Angstrom scale structure. The developed theory provides an understanding of the physical processes underlying the behaviors observed in experiment and simulation and suggests new strategies for enhancing selective polymer membrane design.

## Abstract 2

The molecular size-dependent isothermal penetrant diffusivity in polymer networks is controlled by the cross-link fraction-dependent structural relaxation time and vitrification temperature.

## Introduction

The permeation of atoms, molecules, and nanoparticles of variable size, shape, and chemistry (referred to here as “penetrants”) through a dense polymeric media (liquid, glass, cross-linked permanent or dynamic rubber network, thermoset) is a rich fundamental scientific problem that is broadly relevant in materials, polymer, physical, and biological chemistry.^1−14^ Understanding how the many physiochemical factors and thermodynamic state control penetrant activated mass transport is critical in many applications including gas, water, and organic molecule separations in membranes;^2,8,9,11,15−18^ self-healing based on microcapsules;^19−21^ ion and solvent transport in biological and polymeric materials;^9,22,23^ barrier materials for coatings;^20,21,24^ drug delivery;^5,25^ and nanofiltration.^26,27^ In particular, it underlies a massive chemical separations industry traditionally based on costly and often inefficient distillation methods estimated to constitute 10–15% of the world’s energy consumption.^17^ This has motivated an intensive search for alternative membranes that filter molecules based on their size, shape, and/or interactions with the matrix. A common paradigm is to control the size of membrane pores based on materials such as metal organic frameworks, covalent organic frameworks, and zeolites.^28−31^ Yet, these materials are typically mechanically brittle and difficult to process and deploy at scale. Glassy amorphous polymers are more viable for gas separations,^2,32,33^ and atomistic simulations and phenomenological theoretical models^34−40^ have demonstrated the importance of small amplitude vibrational and torsional polymer motions on activated penetrant transport. Interesting chemical structure-dependent trade-offs between permeability and diffusion have also been identified for these systems.^41^ However, glassy polymer matrices are less size-selective, and ineffective for separations of larger molecules such as polyaromatics which are excluded in such low “free volume” solid materials. Rubbery membranes exploit equilibrium solubility as a selectivity strategy,^42^ but transport is relatively fast and differences in solvation are generally not as sensitive to molecular structure as in materials designed to exploit the activated nature of penetrant diffusion (e.g., polymer glasses). Overall, a major conceptual bottleneck remains due to our still-nascent understanding of the relationship between molecular transport, thermodynamic state, and the chemical structure of the separating species and the polymeric membrane.

Here, we consider nongaseous molecular penetrant diffusion in solvent-free, permanently cross-linked polymer networks. Even in the dilute penetrant limit, understanding how molecular aspects of the polymer control the absolute and relative rates of penetrant diffusivity remains open since activated processes in condensed media can be exceptionally sensitive to a multitude of local physical and chemical factors that characterize the penetrant and polymer species. Recent theoretical work for polymer melts has predicted^43^ that, in the supercooled regime and approaching the glass transition temperature (*T*_g_), the sensitivity of penetrant hopping to polymer structural relaxation is strongly amplified. This insight is qualitatively consistent with early works^34−40^ that found that small-scale motions of the polymeric matrix facilitate the diffusion of small penetrants. Exploitation of this behavior can be an effective means for developing new selective membranes, which we hypothesize can be even further improved if polymer melts are covalently cross-linked.

Our main goal is to study and understand molecular penetrant diffusion in the dilute limit in supercooled cross-linked polymer networks down to *T*_g_. Chemical cross-links induce additional dynamical constraints on penetrant diffusivity via two *qualitatively* distinct processes: (i) slowing down of nanometer-scale polymer segmental relaxation in a highly temperature-dependent manner and (ii) entropic (virtually temperature-independent) geometric mesh obstruction of penetrant diffusion, physical effects which are both enhanced with increasing cross-link density. Here we clarify, for the first time, the competition and inter-relationship between these two effects on penetrant transport over a wide range of temperatures, network mesh sizes, and penetrant sizes. This is a major challenge that classic phenomenological models^9,22,44−47^ cannot address. Moreover, most prior discussions of mesh obstruction effects are based on rigid frameworks or theoretical models for larger nanoparticles in a high-temperature rubber.^48−50^

Our results are of broad relevance toward addressing the physicochemical principles underlying points i and ii above and are based on a coordinated experimental, simulation, and theoretical effort. By experimentally selecting penetrant molecules of variable size and shape within a class of aromatics with similar dispersive (weak) attractions with the polymer matrix, we address how chemical cross-links and activated segmental relaxation determine the penetrant mass transport rate. The simulation- and theory-based modeling purposefully ignore chemically specific penetrant-polymer attractions and adopt coarse-grained spherical penetrant and polymer network models that average over Angstrom scale details. This simplification is motivated not only by practical computational and theoretical considerations, but also by our desire to understand if such fine chemical details are strongly self-averaged in the determination of long-time penetrant diffusion constants. Encouraging support for this concept has been recently established based on a combined theory–experiment analysis^51^ of a large set of atomic and molecular penetrant diffusion data in chemically diverse molecular and polymer liquids over a broad range of temperatures down to the glass transition. Comparison of the new experimental data in cross-linked networks presented here with our theoretical and simulation results shows favorable agreement. This allows us to draw multiple important conclusions that illustrate the generality of the competing physical mechanisms, which will be of major value for the experimental design of selective membranes and the usefulness of simulation and theory for the penetrant transport problem.

## Materials and Methods

All technical details of the experiment, simulation, and theory can be found in the Supporting Information (SI) and our prior paper.^52^ Here we sketch the essential elements.

**Figure 1:**
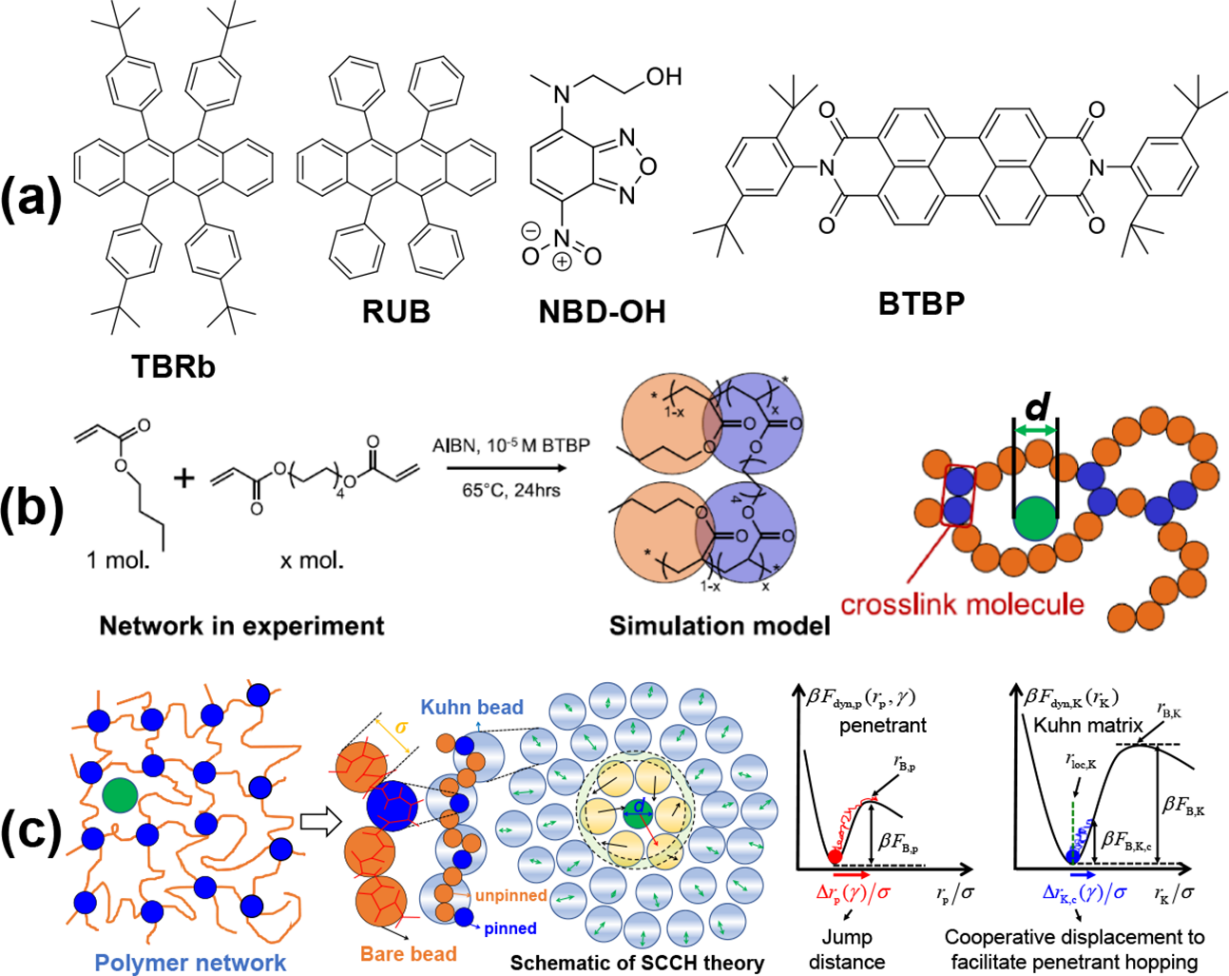
(a) Chemical structures of the penetrants studied. (b) Reaction mechanism for creating the experimental PnBA networks^14,52^ and the corresponding coarse-grained simulation model^52^ where the cross-link (normal) segment is colored blue (orange). (c) Schematic (from left to right) of the polymer network model employed in the theory which is based on the introduction of cross-links via the regular pinning of beads along the chain,^52^ how polymers are coarse-grained to the Kuhn segment scale,^43,52,66^ and the physical ideas of SCCH theory^43,71−73^ based on coupled dynamic free energies for the penetrant and Kuhn segment displacements with a trajectory coupling parameter defined as γ = Δ*r*_p_(γ)/Δ*r*_K,c_(γ) and relevant length and energy scales indicated. Note that although the normal and cross-linked beads are colored the same as those in panel b, the beads or interaction sites of the semiflexible chain model are not identical to the meaning of a bead in the simulation model.^52^

Using fluorescence recovery after photobleaching (FRAP), we experimentally measured at 23 °C the diffusion constant of four penetrant dye molecules [*N*,*N*′-bis(2,5-di-*tert*-butylphenyl)-3,4,9,10-perylenedicarboximide (BTBP), *tert*-butylated rubrene (TBRb), rubrene (RUB), and hydroxy functionalized nitrobenzoxadiazole (NBD–OH)] of differing shapes and sizes (Figure 1a) under dilute conditions in previously well-characterized^14^ dense poly(*n*-butyl-acrylate) (PnBA) polymer networks (schematic of synthesis in Figure 1b; cross-link fraction *f*_cross_). Emission spectra and differential scanning calorimetry and dynamic mechanical analysis data are given in the SI (Figures S1–S3).

The penetrant van der Waals volumes were calculated using an atomic group contribution method^53,54^ (Table 1). To isolate the effect of molecular shape, BTBP (rodlike with aspect ratio ∼3.5) and TBRb (more rounded and platelike) were selected since they have nearly identical space filling volumes. To consider the penetrant volume effect at roughly similar shape, we studied TBRb and RUB. The NBD–OH molecule was examined since it is more spherical than the other penetrants studied, has a significantly smaller volume, and contains a positive and negative charge distribution on the nitro group that is absent in the nonpolar TBRb, RUB, and BTBP penetrants.

**Table 1:** Molecular Volume and Effective Diameter of the Four Penetrants Studied^a^

	BTBP	TBRb	RUB	NBD–OH
*V*/Å^3^	735.88	775.72	493.64	183.7
*d*/Å	11.2	11.4	9.81	7.05
*d*/σ	1.09	1.11	0.95	0.69

a van der Waals volume *V* of the four penetrants investigated^53,54^ and their corresponding diameter *d* if treated as a sphere of equivalent volume.^51^ Penetrant-to-matrix size ratio computed based on σ = 1.03 nm evaluated using *l*_K_ = 2*l*_p_ – σ, where the Kuhn length of PnBA melts is *l*_K_ = 1.72 nm and the ratio of the persistence length to bare bead size in the theory is *l*_p_/σ = 4/3.

Standard molecular dynamics (MD) simulations were performed using our previously validated coarse-grained model for PnBA networks (semiflexible chains^55−60^ where each bead represents a single nBA monomer of size σ*) with explicit cross-links as the polymer matrix in which dilute spherical penetrants (diameter *d*) are dissolved (Figure 1b).^45,47,49,50,61^ The network mesh size is computed as the average distance between cross-link points and the penetrant diffusion constant extracted from the long-time limit of its mean square displacement; see, e.g., Figure S4, and the SI for other technical details of the simulation.^62−65^

Here we generalize to cross-linked networks the microscopic self-consistent cooperative hopping (SCCH) statistical mechanical theory of the activated spherical penetrant (diameter *d*) relaxation time previously developed for polymer melts.^43^ The approach self-consistently predicts the variable degree that smaller scale polymer segmental motions are coupled with the penetrant activated hopping event. Figure 1c presents a schematic of the physical ideas that are based on coupled dynamic free energies for the penetrant and polymer matrix that define the forces on a moving penetrant and Kuhn segment (length *l*_K_), respectively, a priori constructed from knowledge of the microscopic structure. The penetrant activated barrier hopping time and the Kuhn segment correlated motion are self-consistently determined.^43^Figure 1c also indicates how the polymers are coarse-grained to a tangent semiflexible chain of hard-core beads (diameter σ) with parameters that model PnBA, with network cross-links (fraction *f*_n_) modeled as regular pinning of immobilized beads along a chain.^52^ All intermolecular interactions are hard-core repulsions, and the structural correlations required to quantify dynamical constraints are computed using the polymer reference interaction site model integral equation theory.^43,52,66^ The hard-core model is related to thermal networks at 1 atm by a well-tested mapping procedure^52,67,68^ using PnBA experimental equation-of-state data.^51,52,69^ The mean network mesh diameter is calculated as *a*_*x*_ = σ/*Af*_cross_^1/2^ where *A* is calibrated for the PnBA networks and a linear connection between *f*_n_ and *f*_cross_ applies (see the SI and ref (52)). The penetrant alpha process involves hopping over a local cage barrier (*F*_B,p_) that is coupled with longer-range collective displacements of all Kuhn segments outside the cage characterized by an elastic barrier (*F*_el,p_). The total activation barrier is the sum of these two barriers reflecting the coupled local–nonlocal nature of the penetrant relaxation process. The theory predicts the penetrant alpha time as a function of cross-link density, penetrant-to-matrix size ratio (*d*/σ), and temperature. SCCH theory does not explicitly account for mesh confinement effects on penetrant diffusivity.

Prior quantitative comparisons of the segmental alpha time results for the PnBA networks from theory, simulation, and experiment show excellent agreement.^52^ Quantitatively different coarse-graining methods are used corresponding to bare bead sizes of σ* = 0.573 nm in simulation^52^ and σ = 1.03 nm in theory, per Table 1. Both models adopt the experimental PnBA Kuhn length *l*_K_ = 1.72 nm.^70^ The temperature mapping in the theory quantitatively differs from how scaled temperature is converted to absolute temperature in the coarse-grained simulations.^52^

## Conceptual Background and Overview of Core Results

### Background

Activated penetrant diffusivity in cross-linked networks is influenced by both polymer segmental dynamics and entropic geometric mesh confinement. In the qualitative, *but distinct* (see below), spirit of prior work,^48−50^ these two factors for a spherical penetrant of diameter *d* enter its diffusion constant in a multiplicative manner as
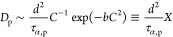
1Equation 1 relates penetrant diffusivity to a product of the rate of its *cross-link fraction* (*mesh size*), *size ratio*, and *temperature-dependent* elementary hopping rate, 1/τ_α,p_ (the focus of SCCH theory), and a temperature-*independent* factor, *X*, associated with an entropic barrier for penetrant traversal through a polymer mesh. The latter depends on the degree of cross-linking quantified by a confinement parameter, *C* = *d*/*a*_*x*_, and the parameter *b* is a chemistry specific numerical factor originally^48,49^ suggested to equal unity corresponding to *X* ≡ exp(−*C*^2^)/*C*. Prior theoretical analysis^48,49^ addressed nanoparticle diffusion in rubbery cross-linked networks at a fixed high temperature in a regime with *C* > 1, very different from the present work. For such systems, it is *not* the penetrant activated alpha time that enters as in eq 1, but rather the *penetrant-independent* Rouse relaxation time of a polymer network strand which depends differently on temperature than the penetrant relaxation time predicted by SCCH theory. The expression *CD*_p_ ∝ exp(−*bC*^2^) does capture well simulations^49^ for dilute spherical nanoparticles in *semidilute* high-temperature rubbery polymer networks with a value of *b* greater than unity (indirectly suggesting the importance of τ_α,p_), despite the fact that *C* greater than unity is not strictly obeyed for some of the simulated systems. In contrast, the experimental, simulation, and theory works reported here for molecular penetrants in dry supercooled polymer networks indicate a different conclusion: cross-linking fraction-dependent segmental relaxation is the most important process for activated penetrant diffusion. More generally, from a physical perspective using τ_α,p_ in eq 1 seems appropriate since mass transport is initiated by a penetrant local hopping event.

For molecular transport, the confinement parameter *C* often does not exceed unity. This motivates us to briefly consider in the SI an alternative model of the effect of network meshes perhaps germane to such a weak (*C* less than unity) confinement regime:^50^
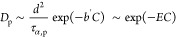
2Here *b*′ and *E* are constants that are expected to depend on the specific penetrant–polymer pair in a manner not quantitatively a priori predicted by existing models. The mean penetrant hopping time τ_α,p_ is taken to be the same as that in eq 1.

Our goal is to determine the relative and absolute importance of polymer segmental relaxation and confinement mesh obstruction on penetrant transport over a wide range of conditions. Unavoidably, directly separating the importance of the latter two factors from experimental or simulation measurements of *D*_p_ is problematic. This is addressed using SCCH theory since it lacks the mesh effect, but it also can be included post facto per eqs 1 and 2. Before presenting our detailed results, we provide a high-level summary of our primary new scientific findings.

### Overview of Core New Findings

The favorable comparison of our experimental data with our theoretical and simulation results allows us to draw the following five important conclusions.

(1) The distinct physical effects of activated segmental relaxation and network mesh confinement affect activated penetrant diffusion to varying degrees. However, our experimental and simulation results can be *empirically described* from either a locally activated penetrant hopping perspective where *T*_g_/*T* is the key variable and an “effective” Arrhenius behavior applies, *or* where the entropic mesh confinement parameter *C* is the key variable. A theoretical understanding of this surprising “degeneracy of interpretation” is constructed. From a causality perspective, we find that the cooling and cross-linking-induced slowing down of segmental relaxation is the dominant factor.
(2) At fixed temperature, the penetrant diffusion constant decreases with its size as an inverse power law with a large exponent of nonhydrodynamic origin.
(3) The good agreement of experiment with simulation and theory based on coarse-grained penetrant and polymer models indicates that local chemical detail even in tightly cross-linked networks is largely “self-averaged” in the determination of the long-time penetrant diffusion constant for the experimental systems we have studied.
(4) Our validated SCCH theory allows one to disentangle the relative importance of the 3 different physical contributions to the activation barrier for molecular transport: (i) local caging by polymer segments in the first solvation shell of the penetrant, (ii) longer-range small amplitude collective elastic displacements of polymer segments farther from a hopping penetrant, and (iii) network mesh confinement. Effects i and ii are intimately coupled in SCCH theory, but for small enough penetrants and/or hot enough polymer networks, the theory predicts the local caging effect (i) is dominant.
(5) The theory makes new testable predictions in a more deeply supercooled regime and/or for larger penetrants not probed in our present experiments or simulations. For example, the rate of penetrant transport becomes even more sensitive to changes of temperature and size ratio due to stronger coupling of penetrant motion with polymer collective elasticity, a mechanism which holds promise for enhancing the selectivity of polymer membranes.

## Results and Discussion

Below we present our experimental, simulation, and theoretical results for penetrant diffusivity in the context of four core issues: (a) role of temperature and cross-linking fraction-dependent polymer segmental relaxation, (b) role of penetrant-to-matrix size ratio, (c) role of entropic mesh confinement, and (d) mechanistic theoretical understanding.

### Role of Segmental Relaxation

**Figure 2:**
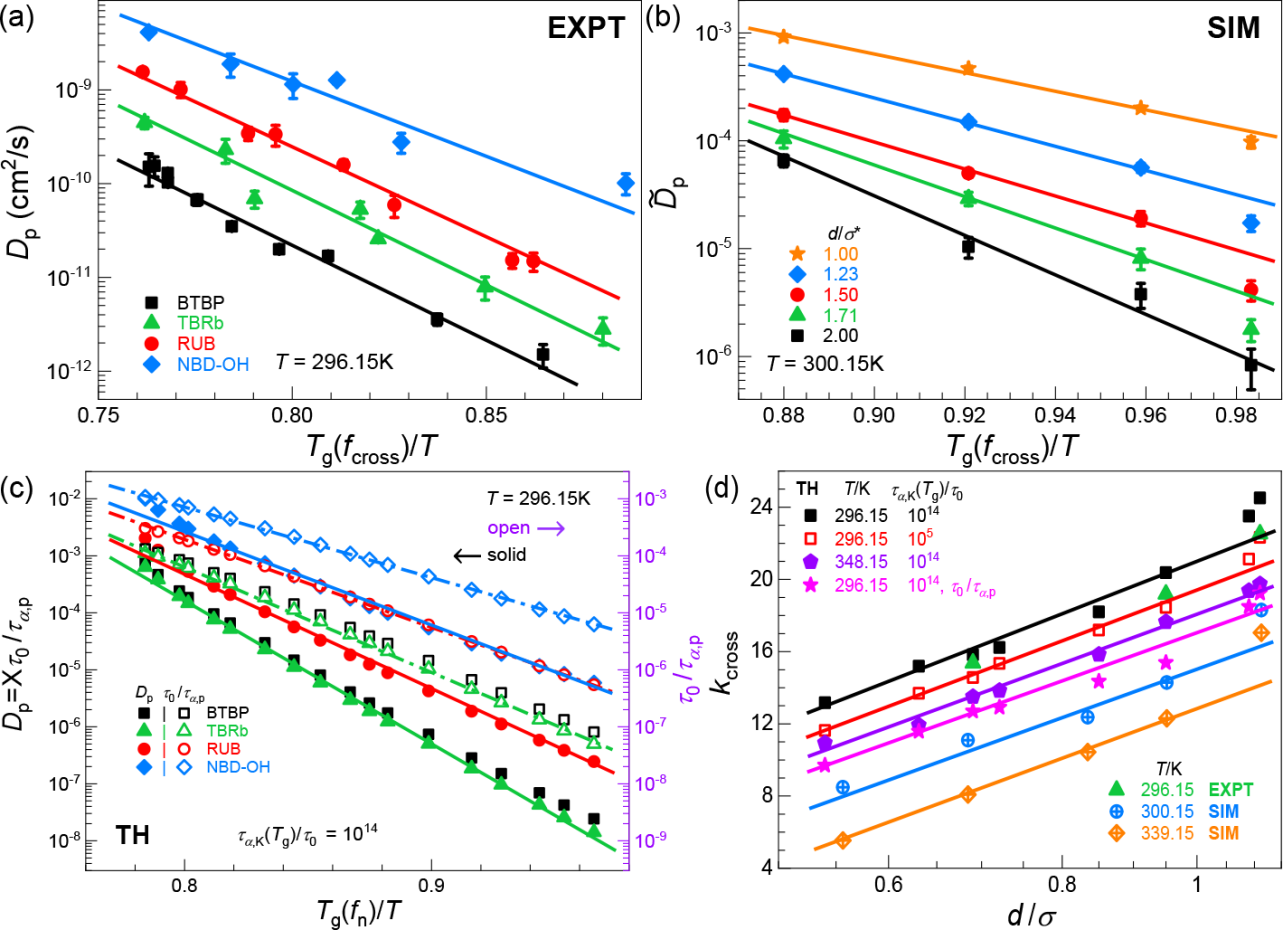
Relationship between penetrant diffusivity and the cross-linking fraction-dependent network glass transition temperature at a fixed temperature. (a) Log–linear plot of the experimental (EXPT) diffusion constants as a function of *T*_g_(*f*_cross_)/*T* over a wide range of cross-link fractions at *T* = 296.15 K (23 °C). (b) Same as in panel a but from simulation (SIM) for 5 penetrants of variable diameters at *T* = 300.15 K. (c) Same as in panel a but for the theory (TH) with penetrant size ratios that mimic the experimental systems per Table 1; the theoretical *D*_p_ (solid symbols with solid lines) is determined using eq 1. Also shown is the predicted penetrant hopping rate, i.e., inverse alpha time (open symbols with dash-dot lines, in units of τ_0_ corresponding to 1 ps per typical viscous liquids), with its *y*-axis indicated on the right-hand side colored in purple and over the same number of decades as that of the left *y*-axis. The *T*_g_ criteria for EXPT, SIM, and TH in panels a–c are the thermodynamic *T*_g_ (see Table S1), τ̃_α,K_(*T*_g_) = 10^5^, and τ_α,K_(*T*_g_)/τ_0_ = 10^14^, respectively. (d) Apparent dimensionless slopes *k*_cross_ deduced from EXPT, SIM, and TH log(1/*D*_p_) versus *T*_g_(*f*_n_)/*T* plots, as a function of size ratio *d*/σ in a linear–log representation. Simulation and theory results for a higher temperature are also shown to support the robustness of our conclusions drawn from experiments performed at a single temperature. The corresponding slopes for the log(τ_α,p_/τ_0_) versus *T*_g_(*f*_n_)/*T* theoretical results are also shown. We note that, in the simulation^52^ (theory), the bead size σ* = 0.573 nm (σ = 1.03 nm, see Table 1), and thus, for comparison purposes, the *x*-axis of the simulation results in panel d is scaled by a factor of 0.573/1.03.

Motivated by the hypothesis that the increase of the polymer segmental relaxation and *T*_g_ with cross-link density is the dominant factor that determines the penetrant diffusion constant, Figure 2a plots the experimental data as a function of *T*_g_(*f*_cross_)/*T* at 23 °C (293.15 K). This choice is motivated by our previous finding that use of the variable *T*_g_(*f*_cross_)/*T* collapses experimental, simulation, and theoretical measures of the segmental relaxation time in polymer networks^52^ over a wide range of cross-link fractions and temperatures. At a fixed temperature, all penetrants show an exponential dependence on *T*_g_(*f*_cross_). The diffusion coefficient at fixed *T*_g_(*f*_cross_) decreases with increasing probe volume from NBD–OH to RUB and TBRb. Note that BTBP exhibits slower diffusion by a factor of 2–3 compared to TBRb despite having almost the same volume, although their apparent dimensionless slopes are nearly identical. This indicates a modest effect of the molecular shape on mass transport and the relationship between absolute diffusivity and *T*_g_(*f*_cross_)/*T*. In the context of the apparent dimensionless slopes *k*_cross_, the dominant factor is the penetrant size, as seen in Figure 2a where the RUB and NBD–OH systems have different slopes. Figure 2d presents the slopes of the data in Figure 2a–c as a function of the size ratio from experiment, simulation, and theory, respectively. Note that the theory and simulation results analyze size ratios germane to our experimental systems. The linear–log plot in Figure 2d establishes an interesting power law behavior between the penetrant diffusion constant and the penetrant-to-matrix size ratio if combined with the linear behavior observed in Figure 2a.

Figure 2b,c shows the corresponding simulation and theory results, respectively. Both capture the basic experimental trends, which we find are robust to increasing temperature beyond that studied experimentally (Figure S6). This level of agreement seems remarkable given penetrants are modeled as spheres and the polymers are coarse-grained. The agreement also provides support for the idea that the penetrant-to-segment size ratio is the most important variable, as recently demonstrated for a wide range of chemically diverse penetrants in molecular and polymer melts.^51^ Of course, the use of a spherical penetrant model does not account for the modest differences between the absolute values of the diffusivity of BTBP and TBRb observed experimentally, although importantly, it does capture the temperature dependences. Moreover, our coarse-grained model may also need to be revisited when penetrant–matrix interactions become strong or directional. We note that, based on other theoretical models^48−50^ that assume that entropic mesh confinement effects dominate the penetrant diffusivity with a cross-link fraction-*independent* prefactor rate, the results in Figure S6 would be the same as that of Figure 2b,c since the confinement parameter is temperature-independent. However, significant differences at different temperatures exist (especially the dimensionless slopes per Figure 2d and absolute values), supporting the idea that activated segmental relaxation (via τ_α,p_ in eqs 1 and 2) is of major importance for penetrant diffusivity. We note that although the variable *T*_g_(*f*_cross_)/*T* has been used in Figure 2a–c and Figure S6, our focus in making such plots is the effects of cross-linking, i.e., the *T*_g_ dependence. Hence the linear relationship of log(*D*_p_) versus *T*_g_(*f*_cross_)/*T* does not imply that an inverse (Arrhenius) temperature dependence of log(*D*_p_) is established.

A potentially important difference between simulations and the experiments and theory predictions is the disparity in time scales probed: the simulation *T*_g_ is defined when *τ*_*α*_(*T*_g_) ≈ 100 ns, dramatically shorter than those in experiment and theory. Nevertheless, the overall good agreement between simulation, theory, and experiment can be understood from our prior work on neat polymer networks,^52^ where it was demonstrated that the vitrification criteria do not significantly change the *normalized T*-dependence or *T*_g_-dependence of the network alpha time *in* the weak and moderately supercooled regimes. Moreover, the inset of Figure S6 confirms that the *T*_g_-dependence of the penetrant diffusivity similarly remains unchanged with the vitrification criteria adopted.

SCCH theory allows one to separate the effects of activated segmental relaxation and mesh confinement on penetrant mobility in networks. This is done in Figure 2c by plotting the theoretical *D*_p_ with and without the factor of *X* in eq 1. Both sets of theoretical results are consistent with an exponential dependence on *T*_g_/*T*, with the *T*_g_-dependence of *D*_p_ computed including the factor *X* modestly stronger than that computed if the mesh obstruction factor is ignored. This quantitative analysis suggests mesh effects are of minor importance for the penetrants and polymer networks studied relative to the consequences of cross-linking-induced slowing down of polymer segmental relaxation.

A further consistency check between experiment, simulation, and theory is to examine the slope of log(1/*D*_p_) versus *T*_g_/*T* in Figure 2a–c, defined as *k*_cross_. Figure 2d shows that it increases with size ratio in an interesting logarithmic manner. Moreover, the experiments (green triangles) are consistent with theory and simulation for very different vitrification criteria. This is true even for the polar molecule NBD–OH that presumably interacts with the polymer in a more chemically specific manner. This further confirms that penetrant size is key in determining the activated diffusion of penetrant, and supports our hypothesis that Angstrom-scale chemical structural features are significantly self-averaged in the long-time diffusivity, at least for the system studied here. At a finer quantitative level, simulation does underpredict the magnitude of *k*_cross_, presumably due to the specific coarse-grained model employed and/or the fact that it does not probe the more deeply supercooled regime germane to experiment. Theoretical predictions for τ_0_/τ_α,p_ that neglect the mesh confinement contribution (no factor of *X* in eq 1) lead to modestly smaller values of *k*_cross_ that deviate more from experiments. Interestingly, one observes that the various results from the *k*_cross_ versus *d*/σ plots determined from τ_α,p_/*τ*_0_ data are parallel to their analogue deduced from 1/*D*_p_. This indicates that the mesh confinement contribution *X* = exp(−*C*^2^)/*C* to *k*_cross_ is effectively independent of *d*/σ to leading order. This can be understood as a consequence of the barrier from entropic confinement, i.e., the slope of the −log(*X*) ∼ *C*^2^ versus *T*_g_(*f*_n_)/*T* plot, changing only weakly (by a factor of ∼3.5–4.7) since we find *C*^2^ ∝ *T*_g_(*f*_n_) based on the theoretical calculations and experimental data shown and discussed in Figure S5.

Although changing temperature is not our primary focus, the magnitude of *k*_cross_ at elevated temperatures has been examined using simulation and theory in Figure 2d. In both cases, heating leads to smaller values of *k*_cross_ due to the reduced barrier for local penetrant hopping. This finding relates to the recent combined theory–experiment work in polymer melts, which established^51^ that the dependencies of the penetrant diffusion constant on size ratio and temperature are nearly independent (barrier factorization), and the temperature dependence is determined by properties of the pure polymer matrix. Thus, at different temperatures, the dependence of *k*_cross_ on penetrant size is not expected to change, as confirmed in Figure 2d by the parallel behavior of the data at different temperatures.

### Role of Penetrant to Polymer Segment Size Ratio

**Figure 3:**
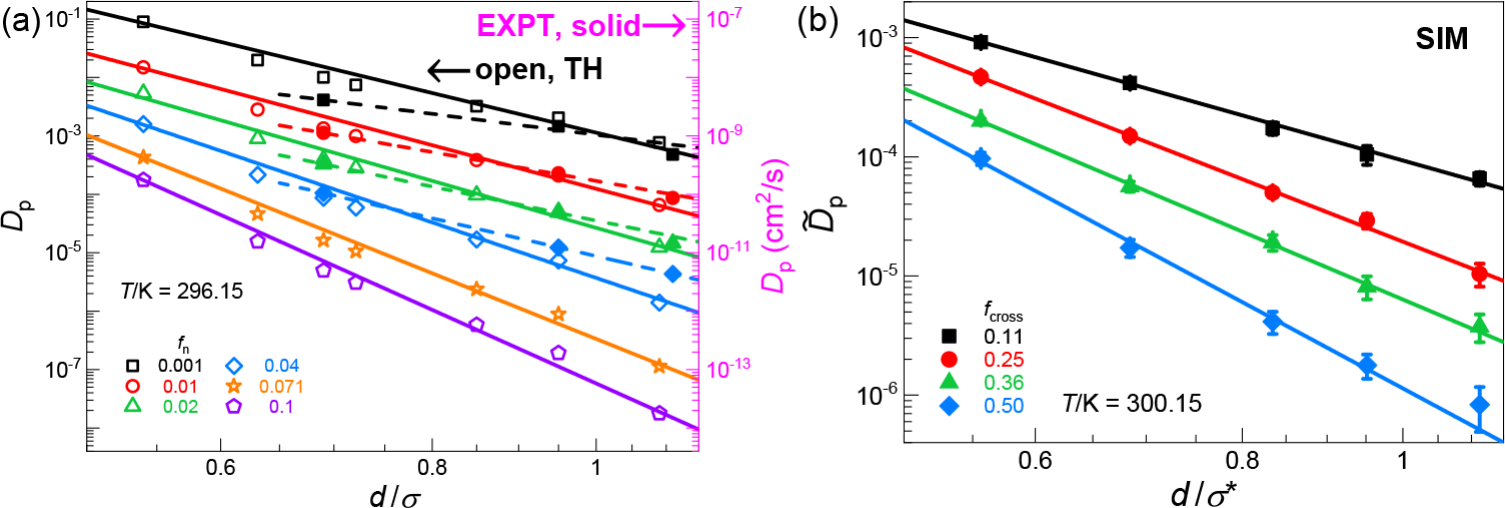
Penetrant diffusivity at various fixed cross-link densities as a function of penetrant-to-matrix size ratio *d*/σ plotted in a log–log manner. EXPT data in panel a is the filled solid symbols with its *y*-axis indicated on the right-hand side colored in pink and over the same number of decades as that of the left *y*-axis. (a) TH (open symbols) and EXPT results are shown at *T* = 296.15 K, and (b) SIM results are shown at *T* = 300.15 K. A summary of the slopes from EXPT, SIM, and TH is given in Figure S8. As in Figure 2d, for the *x*-axes, the SIM value of the size ratio variable is multiplied by the factor 0.573/1.03.

Figure 3 presents theory and simulation results that establish how *D*_p_ varies with size ratio over a wide range of cross-linking fractions at a fixed temperature. This aspect is relevant to the problem of isothermal selectivity of penetrant transport based on the size. Given our findings above that log(1/*D*_p_) ∝ *T*_g_(*f*_cross_) and the proportionality constant *k*_cross_ is a logarithmic function of size ratio *d*/σ, we conclude that (i) probe diffusivity decreases as a power law with *d*/σ, and (ii) activated segmental relaxation dominates the penetrant size dependence. Interestingly, finding i is qualitatively the same as found in prior SCCH theory analysis for dilute spherical penetrants in semiflexible polymer chain melts^43,51^ which revealed that a power law relation applies well over a wide range of *d*/σ (including the large size ratio regime) and which remains robust even for systems containing weak attractions between the penetrant and matrix.^43^ Here we find that when cross-linking is introduced, the predicted power law relation between 1/*D*_p_ and *d*/σ still works very well for fixed cross-link density, although slight quantitative deviations are noticeable in Figure 3a [see also Figure S9c where log(*d*/σ) is used as *x*-axis]. Moreover, we find that the exponent of the power law [i.e., the slope *k*_size_ of the log(1/*D*_p_) versus *d*/σ plots] varies *linearly* with cross-link fraction *f*_n_ (see Figure S8) to within slight uncertainties. This interesting result can be understood by combining our above findings that log(1/*D*_p_) ∝ *T*_g_(*f*_cross_) and *C*^2^ ∝ *T*_g_(*f*_n_) with the definition of confinement parameter (*C* ∼ *f*_cross_^1/2^), as shown in the SI.

Overall, the SCCH theory calculations performed at the experimental measurement temperature agree well with the corresponding experimental and simulation results in Figure 3a,b, respectively. Moreover, the predicted trends remain robust at elevated temperatures (Figure S10c,d). We note in passing that the *k*_cross_ versus *d*/σ plot also exhibits good linearity (Figure S7b). When this result is combined with our finding that log(1/*D*_p_) ∝ *T*_g_(*f*_cross_), an alternative linear relationship between log(*D*_p_) and *d*/σ (an exponential, not power law, dependence) is obtained, although it is valid over a more limited lower *d*/σ range (see Figure S9a,b), as expected.^43,72,73^

### Confinement Mesh Perspective

Our analysis in the prior two subsections was motivated by the idea that the key factor in the determination of the penetrant diffusion constant is the cross-link-induced enhancement of its local activated hopping time. We now explore in detail the very different limiting perspective (per eqs 1 and 2) that the entropic mesh confinement effect can empirically “explain” our results.

We first consider the theoretical results from an analytical perspective. Surprisingly, a “mesh-confinement-like” form can be obtained from an activated segmental dynamics analysis. This follows by combining the results log(τ_α,p_/τ_0_) ∝ *T*_g_(*f*_n_) and *C*^2^ ∝ *T*_g_(*f*_n_) discussed above and in the SI, which implies that the theory predicts log(τ_α,p_/τ_0_) ∝ *C*^2^. Hence, a *noncausal* “degeneracy of interpretation” at fixed temperature is predicted (not assumed) by the theory. Its origin is that cross-linking modifies the local penetrant hopping activation barrier in a manner that depends on the confinement parameter *C* in the same manner as does the entropic mesh mechanism. In reality, an additional contribution to the penetrant barrier from a true entropic mesh confinement effect is potentially important, i.e., *X* = exp(−*C*^2^)/*C* in *D*_p_ = *Xτ*_0_/τ_α,p_ of eq 1, thereby yielding *CD*_p_ ∼ exp(−*BC*^2^), where *B* is a *C*-independent numerical factor.

**Figure 4:**
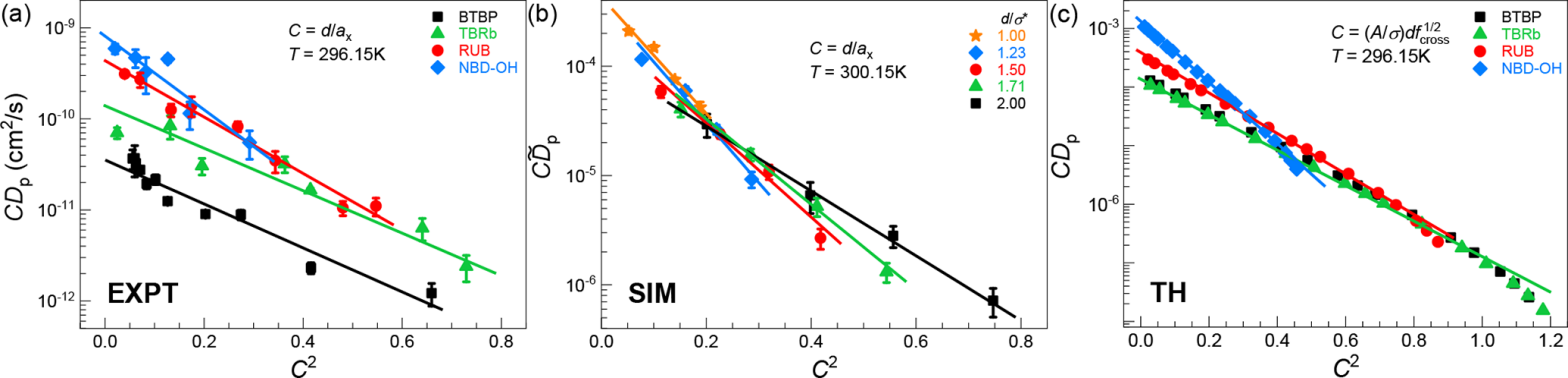
Penetrant diffusivity from an entropic mesh confinement perspective per eq 1. Data for various penetrants are plotted as log(*CD*_p_) versus *C*^2^ for (a) EXPT, (b) SIM, and (c) TH over a wide range of cross-link fractions at a fixed temperature (*T* = 296.15 K for EXPT and TH, and *T* = 300.15 K for SIM). The confinement parameter is *C* = *d*/*a*_*x*_ in EXPT and SIM, and *C* = (*A*/σ)*d*(*f*_n_/0.19)^1/2^ in TH (see the SI). The simulation *C* is again multiplied by 0.573/1.03 to allow comparison to EXPT and TH.

Figure 4 aims to separate the consequences of the two very different physical effects on penetrant diffusivity and further test our new theoretical insights using experimental and simulation data. Linearity between log(*CD*_p_) and *C*^2^ is observed for all cases. However, the apparent slope in Figure 4 decreases with penetrant size, which is the *opposite* trend found by varying penetrant size for the slopes in Figure 2a–c which were constructed based on the very different cross-link fraction-dependent activated segmental dynamics perspective. Our finding in Figure S5 that *T*_g_(*f*_n_) ∝ *C*^2^ was deduced under fixed *d* conditions, and thus, the core insight is *T*_g_(*f*_n_) ∝ *C*^2^/*d*^2^, where *C* = *d*/*a*_*x*_. Applying this proportionality to the above analysis yields log(1/*CD*_p_) ∼ *k*_cross_*T*_g_ ∼ (*k*_cross_/*d*^2^)*C*^2^. Figure 2d shows that *k*_cross_ varies very weakly (logarithmically) with penetrant diameter compared to the quadratic (*d*^2^) dependence in this expression, thereby providing an explanation for why the apparent slopes in Figure 2a–c (*k*_cross_) increase, but those in Figure 4 (*B* ∼ *k*_cross_/*d*^2^) decrease with penetrant size. The theory results in Figure 4c also establish the operational robustness of the relation *CD*_p_ ∼ exp(−*BC*^2^) in the large *C* regime, which is *not* due to entropic mesh confinement but rather to cross-link-dependent penetrant local activated hopping.

We have also tested whether the relation *D*_p_ ∼ exp(−*EC*) with *E* an adjustable constant (per eq 2) can empirically model our data. We find in the “weak confinement” range 0.15 < *C* < 0.9, it works well for experiment, simulation, and theory (see Figure S11). This can be theoretically understood from an activated segmental relaxation perspective since only the local cage barrier is important in this regime, and SCCH theory predicts its scales *linearly* with *C*. Hence, the success of the form *D*_p_ ∼ exp(−*EC*) signals another noncausal “degeneracy of interpretation” of what controls penetrant diffusivity. More generally, by revisiting previous simulation data,^50^ we find that in a practical sense both relations motivated by an entropic mesh confinement dominated perspective (eqs 1 and 2) work rather well in the limited range of *C* analyzed (see Figure S12). However, in a large enough *C* regime we predict based on SCCH theory that such an empirical entropic mesh perspective fails (Figure S11c) due to the growing importance of longer-range collective elasticity of the polymer matrix in determining the penetrant hopping rate. Detailed discussion of these issues is presented in the SI, including the robustness of our new insights at elevated temperatures (see Figure S13).

### Mechanistic Theoretical Understanding

The overall good agreement between experiment, simulation, and theory, despite being performed in nonidentical supercooled regimes, suggests that an intriguing simplicity is at play. In this subsection, the physical mechanism is elucidated based on SCCH theory and our understanding of the penetrant local cage and longer-range collective elastic activation barriers as a function of temperature, size ratio, and cross-linking fraction.

**Figure 5:**
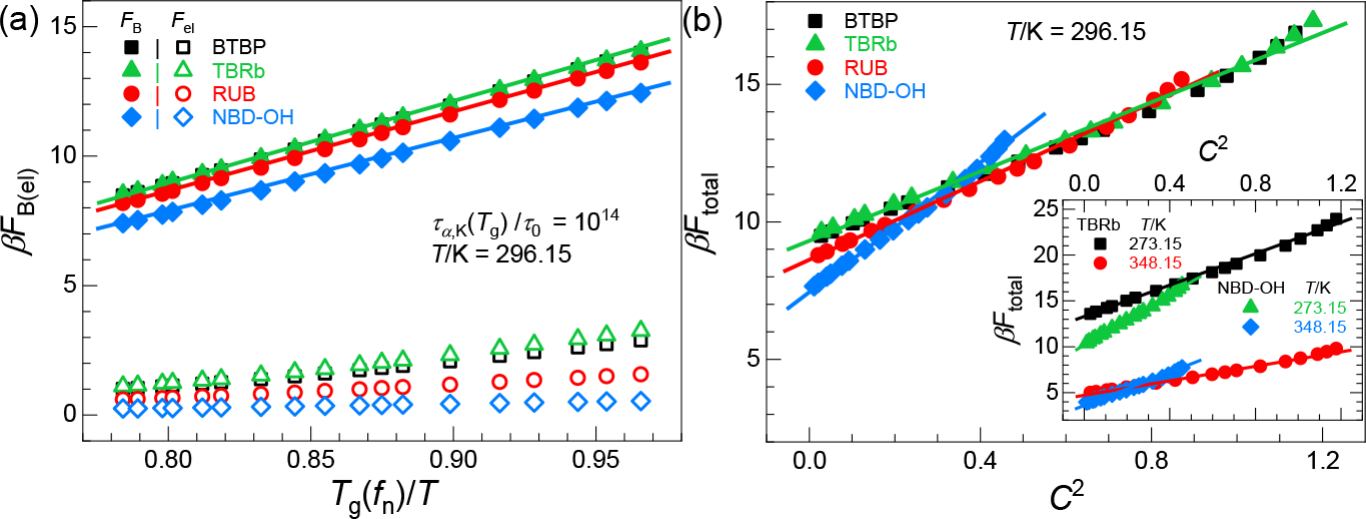
Theory predictions for the penetrant barriers in units of thermal energy. (a) Local cage and collective elastic barriers as a function of *T*_g_(*f*_n_)/*T* over a wide range of cross-link fractions at a fixed *T* = 296.15 K. The very small differences between the elastic barriers of BTBP and TBRb reflect the fact that their sizes are not exactly the same (see Table 1). (b) Same as in panel a but for the total barriers as a function of *C*^2^. Inset: Total barriers for TBRb and NBD–OH as a function of *C*^2^ at lower (*T* = 273.15 K) and higher (*T* = 348.15 K) temperatures.

Figure 5a shows that the collective elastic barriers for all molecular penetrants studied are far smaller than their local cage analogues. Regardless of the precise *T*_g_(*f*_n_) vitrification criteria adopted, *both* the local cage and elastic barriers vary linearly with *T*_g_(*f*_n_)/*T* over the entire *T*_g_/*T* range studied for all of the penetrants. This provides a theoretical explanation of the exponential relationship between the alpha relaxation time or diffusion constant and *T*_g_(*f*_n_)/*T* in Figure 2c. Furthermore, although Figure 5a shows that the local cage and elastic barriers both increase with penetrant size, the rate of growth of the local cage barriers remains nearly independent of penetrant size, while that for the elastic barrier strongly increases. Thus, the slope change in Figure 2c of log(τ_0_/τ_α,p_) versus *T*_g_(*f*_n_)/*T* plots with penetrant size is mainly a consequence of the increasing relevance of the longer-range collective elastic barrier relative to its local cage analogue.

Combining the above results with *C*^2^ ∝ *T*_g_(*f*_n_) discussed above and in the SI, we predict that the penetrant local cage and elastic barriers vary linearly with *C*^2^ (see Figure S14a). This provides an understanding of our numerical finding of a linear relationship between the penetrant total barrier and *C*^2^ seen in Figure 5b and its inset for various penetrant sizes and temperatures. This insight thus establishes the underlying physical reason for the operational usefulness of the relation τ_α,p_/τ_0_ ∼ exp [−(*B* – 1)*C*^2^] or *CD*_p_ ∼ exp(−*BC*^2^) (where *B* is a numerical factor), even under deeply supercooled conditions where the penetrant total barrier is high. This prediction can be tested in future experiments and/or simulations that probe even slower mass transport characterized by higher total barriers (lower temperatures, larger penetrants) than discussed in our present work.

Finally, because penetrant barriers are predicted to grow linearly with *C*^2^, a literal linear relationship between them and *C* does not apply, as confirmed in Figure S14b. However, when *C* is not sufficiently large (0.15 < *C* < 0.9) corresponding to weak or nonexistent mesh confinement, the penetrant local cage barrier does effectively grow linearly with *C* when the collective elastic barrier is negligible. This suggests that 1/τ_α,p_ in eq 2 is proportional to exp(−*b*″*C*), and explains why our experimental and simulation data (and also other literature data^50^) can be empirically fitted by the exponential relation *D*_p_ ∼ exp(−*EC*) over the limited range of *C* typically measured per the last equality of eq 2 (where *E* = *b*′ + *b*″). If in future experiments and simulations the confinement parameter is further increased and/or the temperature is lowered to a more deeply supercooled regime, we predict that the exponential relation *D*_p_ ∼ exp(−*EC*) will eventually fail.

## Conclusions

We have combined experiment, simulation, and theory to unravel the competing effects of penetrant size on its transport in cross-linked polymer networks over a range of cross-link densities, size ratios, and temperatures. The crucial importance of the temperature and chemistry specific degree to which penetrant hopping is coupled to the polymer structural relaxation process has been established, with the entropic barrier due to mesh confinement being of quantitative, but secondary, importance. The leading order effect of network cross-links is to slow down polymer structural relaxation and greatly suppress the elementary penetrant hopping event. However, there are also noncausal “empirical correlations” between the behavior of penetrant transport largely controlled by cross-link-induced slowing down of segmental relaxation and what is expected based on a mesh confinement perspective. The reason for this surprising “degeneracy of interpretation” is understood within the theory as a consequence of a common dependence of the dynamic activation barriers on the confinement parameter *C* and how the entropic obstruction barrier scales with *C*.

The good agreement between experiment, simulation, and theory provides support for the size ratio (penetrant diameter to Kuhn length) as a key variable and more generally the usefulness of coarse-grained models that average over Angstrom scale chemical details in cross-linked networks. The SCCH theory reveals the microscopic physics underlying the behaviors found in experiment and simulation in terms of local cage and longer-range collective elastic barriers to penetrant hopping and how they depend differently on the degree of cross-linking and temperature. Testable predictions in a more deeply supercooled regime not probed in our present experiments or simulations were made, where penetrant transport is predicted to become even more size sensitive. We believe our work illustrates the generality of the physical mechanisms identified which are relevant for selective membrane design, and establishes the usefulness of simulation even at temperatures well above those often probed in experiment.

Of course, the relative importance of the segmental relaxation and mesh confinement effects on penetrant diffusion will depend on temperature (e.g., rubbery vs supercooled regime) and the precise value of the mesh confinement parameter. Further work is needed in this direction, and our analysis provides a roadmap to anticipate and understand how the two primary consequences of chemical cross-linking impact penetrant mass transport. Other major future directions are to apply our combined experiment–theory–simulation approach to address the dynamical impact of penetrant–polymer specific attractive interactions, generalize the simulation and theory approaches to explicitly treat the role of molecular penetrant shape and polymer strand flexibility on transport, and address penetrant diffusion in associating polymer liquids^74^ and vitrimers.^75−78^

## Supporting Information Available

Details of the materials and methods underlying the experiments (fluorescence spectra, DSC curves, and DMA curves of the polymer networks), simulations, and theory; additional theoretical and simulation evidence for the crucial role of network segmental relaxation in determining the penetrant diffusion constant at elevated temperatures, and results for different *T*_g_ criteria to further establish the robustness of our findings; additional simulation and theoretical results for the penetrant-segment size ratio dependence of the penetrant diffusion constant; entropic mesh confinement analyses of the penetrant diffusion constant; and additional discussion of the mechanistic insights deduced from SCCH theory (PDF)
