# Supplementary material for: How Segmental Dynamics and Mesh Confinement Determine the Selective Diffusivity of Molecules in Cross-Linked Dense Polymer Networks
Source: ACS Cent Sci. 2023 Feb 24;9(3):508–18. doi: 10.1021/acscentsci.2c01373 (PMC10037493; doi:10.1021/acscentsci.2c01373)
Supplement: Supplementary file 1 — oc2c01373_si_001.pdf [file oc2c01373_si_001.pdf]

Supplementary Information for "How Segmental Dynamics and Mesh Confinement Determine the Selective Diffusivity of Molecules in Dense Crosslinked Polymer Networks"

Baicheng Mei [a,d], Tsai-Wei Lin [c,d], Grant S. Sheridan [a,d], Christopher M. Evans [*a,c,d],

Charles E. Sing [*a,c,d] and Kenneth S. Schweizer [*a,b,c,d]

[a.] *Department of Materials Science, University of Illinois, Urbana, IL 61801, USA*

[b.] *Department of Chemistry, University of Illinois, Urbana, IL 61801, USA*

[c.] *Department of Chemical & Biomolecular Engineering, University of Illinois, Urbana, IL 61801, USA*

[d.] *Materials Research Laboratory, University of Illinois, Urbana, IL 61801, USA*

[*] *kschweiz@illinois.edu* [*] *cesing@illinois.edu* [*] *cme365@illinois.edu*

For the benefit of the reader, Section I recalls more details of the Materials and Methods underlying the experiments, simulations, and theory work in the main text. Section II provides additional theoretical and simulation evidence for the crucial role of network segmental relaxation in determining the penetrant diffusion constant at elevated temperatures, and results for different T_g criteria to further establish the robustness of our findings. Section III presents additional simulation and theoretical results for the penetrant-segment size ratio dependence of the penetrant diffusion constant. The entropic mesh confinement analyses of the penetrant diffusion constant are further discussed in section IV. Finally, section V presents a more detailed discussion of the mechanistic insights deduced from SCCH theory.

I. Details of Experiment, Simulation and Theory

A. Experiment.

The synthesis of n-butyl acrylate (nBA) networks has been reported in our prior work.[1] In brief, the networks were synthesized via the neat free radical polymerization of nBA and 1,8-octanediacrylate with varying molar ratios from 0.01 to 0.5, using AIBN as the initiator at 65 °C with 10^{-5} M of four different dye molecules dissolved in the monomer. BTBP was the one dye studied previously[1], while the new three dyes of different size and shape are: tert-butyl substituted rubrene (TBRb, >99%, Ossila), rubrene (RUB, 99.99%, Sigma-Aldrich), and propanol substituted nitrobenzofurazan (NBD-OH, synthesized following a literature protocol[2]). The van der Waals volume of each penetrant was calculated using an atomic group contribution method[3, 4]. These values are reported in **Table 1** along with the diameter corresponding to a sphere of equivalent volume. BTBP (aspect ratio ~3.5) and TBRb (more round plate-like shape) were selected in order to isolate the effect of penetrant shape since they have nearly identical volumes (**Table 1**). To consider the effect of different penetrant volumes, while fixing the aspect ratio, TBRb and RUB were used. The fourth penetrant NBD-OH is the most spherical molecule, has the smallest volume, and bears a positive and negative charge on the nitro group.

The emission/excitation spectra for each dye were determined using a Horiba PTI-Quantamaster to determine the concentration where aggregation was no longer a concern (**Figure S1**). Time-dependent illumination of the networks was used to evaluate the bleaching of the dye under similar conditions to the fluorescence (FRAP) experiments. The FRAP procedure for BTBP and analysis method were described in prior work[1]. For RUB, TBRb, and NBD-OH, the FRAP experiments were carried out using a 488nm laser on a Leica SP8 Confocal Microscope. The

networks (~60 μm) were kept in an inert Argon glovebox on a glass coverslip until running FRAP. All measurements were performed at 23 °C on a Peltier temperature-controlled stage.

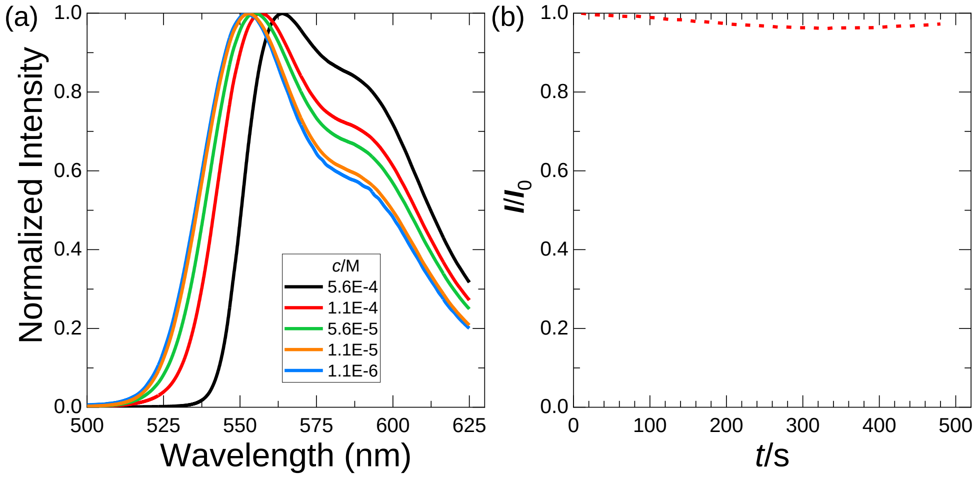

Figure S1. (a) Normalized RUB emission spectra intensity excited at 481 nm at various concentrations, *c* (in unit of M), to determine the dilution needed for FRAP experiments. Below a 10^{-5} M dye concentration the emission spectra no longer changes (or changes very slightly). (b) RUB emission intensity stability at 481 nm excitation on the fluorimeter over 8 minutes showing only 5% bleaching indicating it is appropriate for the FRAP experiments.

The T_g of each polymer network was determined by differential scanning calorimetry (TA Instruments DSC 2500) using a heat-cool-heat ramp protocol at 10 °C/min. The first derivative of the second heating curve was calculated to aid in visualizing the breadth which grows substantially with increasing crosslink density. Each of the dyes were incorporated into the DSC samples to confirm the dye does not impact the macroscopic physics of the networks at such trace levels. **Figure S2** shows the derivative heat flow curves for all networks in this study, and the BTBP samples were previously reported.[1] All networks show an increase in T_g and the T_g breadth with increased crosslinking, and these values are summarized in **Table S1**.

Table S1. Summary of DSC and DMA results for T_g, T_g breadth ΔT_g, N_x, and a_x for the PnBA networks studied with the four types of dyes: (a) BTBP; (b) TBRb; (c) RUB; and (d) NBD-OH.

(a) BTBP

Mol % crosslinker	T_g (°C)	ΔT_g (°C)	N_x	a_x (nm)
1.1	-47	13	92	4.7
1.2	-47	13	85	4.6
1.2	-46	13	82	4.5
1.4	-46	13	71	4.2
1.7	-43	13	59	3.9
2.1	-43	13	48	3.5
2.6	-41	14	38	3.2
4.4	-37	14	22	2.5
6.1	-33	15	15	2.1
9.9	-25	18	9.1	1.7
16.9	-17	21	4.9	1.4

(b) TBRb

Mol % crosslinker	T_g (°C)	ΔT_g (°C)	N_x	a_x (nm)
1.0	-48	11	97	4.9
2.6	-41	11	37	3.1
4.4	-39	13	21	2.5
8.1	-31	13	11	1.9
9.8	-30	13	9.2	1.7
15.7	-22	14	5.4	1.4
21.1	-13	17	3.7	1.3

(c) RUB

Mol % crosslinker	T_g (°C)	ΔT_g (°C)	N_x	a_x (nm)
1.0	-47	11	99	4.9
1.9	-45	11	53	3.7
3.7	-40	11	26	2.7
5.0	-38	12	19	2.3
8.1	-32	13	11	1.9
10.8	-28	14	8.3	1.7
16.0	-19	17	5.3	1.4

18.6	-18	14	4.4	1.3

(d) NBD-OH

Mol % crosslinker	T_g (°C)	ΔT_g (°C)	N_x	a_x (nm)
1.0	-47	10	100	4.9
3.3	-41	11	30	2.8
4.5	-36	11	21	2.5
7.3	-33	12	13	2.0
10.3	-28	13	8.7	1.7
19.3	-11	16	4.2	1.3

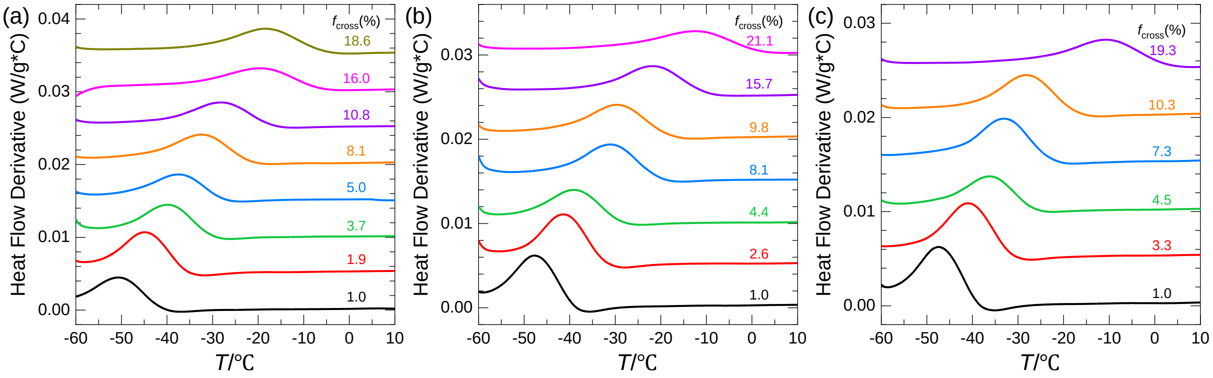

Figure S2. DSC derivative heat flow curves for PnBA networks with added dyes (a) RUB, (b) TBRb, and (c) NBD-OH. The f_{cross} value is shown next to each curve. Dye incorporation does not affect the T_g value or the dependence on crosslink density within errors.

Dynamic mechanical analysis (TA Instruments DMA Q800) was used to determine the elastic modulus of the networks which was then used to calculate the mesh size. Stress-strain curves are shown in **Figure S3**, and all exhibit a *linear* response up to 1.0 % strain. The slope is taken as the Young's modulus, and the presence of trace dye does not affect the value.

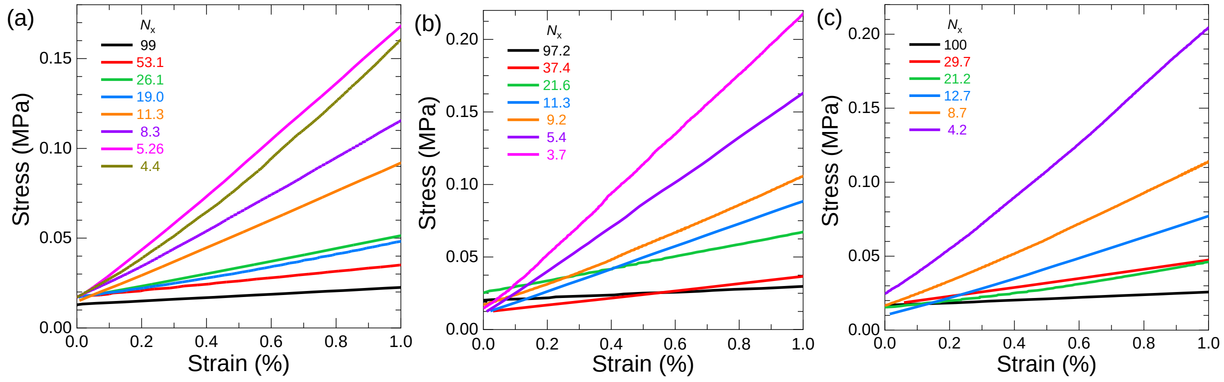

Figure S3. DMA stress-strain curves for networks with (a) RUB, (b) TBRb, and (c) NBD-OH taken at 23 °C under tension. Number average degree of polymerization between crosslinks is shown in the legends.

B. Simulation

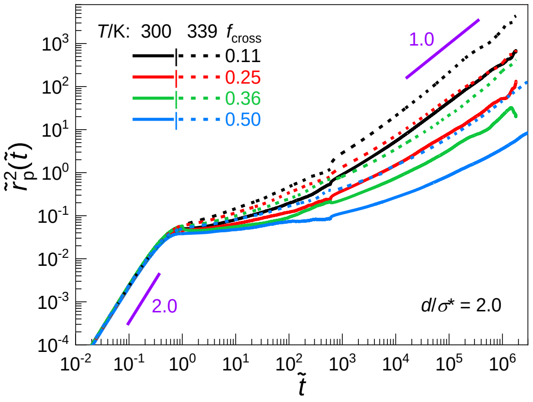

Figure S4: Time evolution of mean-square-displacement of penetrant, $\langle \tilde{r}_p^2(\tilde{t}) \rangle$, in simulation at different values of f_{cross} at a lower (300K) and a higher (339K) temperatures. The penetrant diffusion coefficient is obtained in the diffusive regime (long-time scale) where the time scaling exponent is 1.0.

The simulation model is built upon our previous model for neat networks[5]. Here we briefly summarize our previous model and include simulation details for the penetrant diffusion in networks problem. Dimensionless simulation quantities with tildes (e.g. $\tilde{T} = T/T^*$) are employed to distinguish them from the analogous experimental and theoretical quantities. The following dimensionless variables are employed: bead diameter σ^* for length (different from the σ used in theory), temperature normalized by T^* (the temperature scale determined from parametrization step, see details in Ref.5), energies normalized by $k_B T^*$, and times normalized by $\tau^* =$

$\sqrt{m\sigma^2/k_BT^*}$, where m is the nBA monomer mass. We note that our simulation temperatures are slightly different than those used in experiments. This is the result of parameterization choices for the adopted coarse-grained model made in Ref.5 to describe the segmental relaxation of crosslinked polymer networks, which serves as the foundation for our present new work on penetrant diffusion. We expect the comparison between experiment and simulation to be useful despite this slight 4ºC difference. For context, we note that this is a much smaller temperature difference than what is seen from simulation in **Figure 2d**, which shows results for temperatures that are 40ºC and exhibit similar physical trends.

The initial system is composed of twenty linear chains ($N_c = 20$) with $N_m = 30$ the number of monomer beads per chain and N_p the number of spherical penetrants of diameter $\tilde{d}$ in a cubic box with periodic boundary conditions in three dimensions. Polymers are modeled as standard semiflexible chains[6-11] where each bead represents a single nBA monomer.

The overall energy contains contributions from bonding interactions $\tilde{U}_B$, a bending potential $\tilde{U}_\theta$, and Lennard-Jones interactions $\tilde{U}_{LJ}$:

$$\tilde{U} = \tilde{U}_B + \tilde{U}_\theta + \tilde{U}_{LJ} = \sum_{i>1} \tilde{u}_{B,i} + \sum_{i>1} \tilde{u}_{\theta,i} + \sum_{ij} \tilde{u}_{LJ,ij} \quad \text{(S1)}$$

Here the total system energy is written in terms of the pairwise contributions $\tilde{u}_{B,i}$, $\tilde{u}_{\theta,i}$, and $\tilde{u}_{LJ,ij}$ corresponding to their respective energy contributions. Bonded monomers interact through harmonic bonding potential:

$$\tilde{u}_{B,i} = \frac{\tilde{k}}{2}\left(\tilde{r}_{i,i-1} - 1\right)^2 \quad \text{(S2)}$$

where the large spring constant $\tilde{k} = 2000$ is adopted to enforce the equilibrium distance between the i and $i-1$ monomers as $\tilde{r}_{i,i-1} = 1$.[12-15] A bending energy is introduced to account for chain stiffness:

$$\tilde{u}_{\theta,i} = \tilde{k}_\theta[1 - \cos\theta_i] \tag{S3}$$

where the bending constant $\tilde{k}_\theta = 1.52$ is selected to reflect the experimental Kuhn length of PnBA. Further details of the parametrization is given in Ref.5. A Lennard-Jones (LJ) potential is used to describe all non-bonded interactions,

$$\tilde{u}_{\mathrm{LJ},\alpha\beta} = \begin{cases} 4\tilde{\epsilon}_{\alpha\beta}\left[\left(\frac{\tilde{d}_\alpha+\tilde{d}_\beta}{2\tilde{r}_{\alpha\beta}}\right)^{12} - \left(\frac{\tilde{d}_\alpha+\tilde{d}_\beta}{2\tilde{r}_{\alpha\beta}}\right)^{6}\right], \tilde{r}_{\alpha\beta} < \tilde{r}_{\mathrm{cut}} = 2.5 \times \frac{\tilde{d}_\alpha+\tilde{d}_\beta}{2} \\ 0, \text{ otherwise} \end{cases} \tag{S4}$$

where $\alpha, \beta \in \{\mathrm{n}, \mathrm{p}\}$, with n denoting the monomer bead in the network and p denotes the penetrant, and $\tilde{d}_\mathrm{n}$ is always unity (i.e., $d_\mathrm{n} = \sigma^*$) and $\tilde{d}_\mathrm{p} \equiv \tilde{d}$. In this study, we only consider the effect of size of different penetrants (not specific penetrant-polymer attractions) so that $\tilde{\epsilon}_{\mathrm{nn}} = \tilde{\epsilon}_{\mathrm{np}} = \tilde{\epsilon}_{\mathrm{pp}} = 1$.

The system is first equilibrated at $\tilde{T} = 1$, $\tilde{P} = 0$, and then networks are prepared by crosslinking the linear chains with reactive beads randomly distributed along the chain[16, 17]. The total number of reactive beads is $N_\mathrm{r} = f_\mathrm{r} N_\mathrm{m} N_\mathrm{c}$, where f_r is the fraction of reactive beads which tunes the crosslink density of networks. If the distance between a reactive bead and a free bead (orange bead in **Figure 1b** of the main text) is within $\tilde{R}_{\mathrm{min}} = 1.1$, a new permanent bond will be formed given an assigned probability. Once a reactive bead forms a bond with a free bead, both the reactive bead and the free bead are labeled as crosslink beads (dark blue beads in **Figure 1b** of the main text) and these two beads represent one crosslink 'molecule'. Crosslinking reactions were turned off once every reactive bead has formed a new bond with another free monomer (maximum number of possible bonds has been reached). In the end, N_r reactive beads have reacted with the N_r free beads and they turned into $2N_\mathrm{r}$ crosslink beads and belong to the N_r crosslink molecules.

The crosslink density, f_{cross}, of networks is defined as

$$f_{\text{cross}} = \frac{n_{\text{crosslink}}}{n_{\text{crosslink}}+n_{\text{monomer}}} = \frac{N_{\text{r}}}{N_{\text{m}}N_{\text{c}}-N_{\text{r}}}. \quad \text{(S5)}$$

Here, $n_{\text{crosslink}}$ and n_{monomer} are the number of moles of crosslink molecules and nBA monomers. Four values of crosslink fraction f_{cross} are considered: 0.11, 025, 0.36, and 0.5. The mesh size of networks at different crosslink densities is defined as the averaged distance between two adjacent crosslink beads on the same chain. The glass transition temperature T_{g} is determined from a dynamic criterion. The alpha relaxation time ($\tilde{\tau}_{\alpha}$) at each temperature was defined as the time where the temporal autocorrelation function of a Kuhn monomer vector, $C_{\lambda}(\tilde{t}) = \langle P_2(\tilde{\boldsymbol{r}}_3(\tilde{t}) \cdot \tilde{\boldsymbol{r}}_3(0))\rangle$ decays to $1/e$.[18-22] Here, P_2 is the second Legendre polynomial and $\tilde{\boldsymbol{r}}_3$ is a vector between two beads that are 3 bonds apart which reflects the choice of coarse-grained bead relate to Kuhn segment of PnBA. T_{g} is then defined as when the alpha relaxation time is $\tilde{\tau}_{\alpha}(\tilde{T}_{\text{g}}) = 10^5$. More information can be found in our previous work[5].

After crosslinking, the system is cooled to a target temperature at a cooling rate $\tilde{\Gamma} = 8.3 \times 10^{-6}$ (corresponding to $\Gamma = 1.25 \times 10^9$ K/s in experimental units) and further equilibrated at constant $\tilde{P} = 0$. Another short NPT run was performed and the mean volume V is measured. We then switch to a NVT ensemble by setting the system volume to the mean volume V and equilibrate the system before final production run at NVT.[23] All simulations are performed in LAMMPS[24] with a standard Nosé-Hoover thermostat and extended-ensemble barostat[25-27], and each quantity obtained from simulation is averaged over five independent trajectories.

C. Theory

We here generalize for the first time the SCCH theory of dilute hard sphere (HS) penetrant diffusion in polymer melts[28] to crosslinked polymer networks (see **Figure 1c** in the main text for an illustration of the key conceptual elements). Within this theory[28-31], the activation barrier and corresponding mean hopping time for penetrant motion, and the extent of coupling of its transport

with the early, medium, and late stages of the matrix structural relaxation process, can be predicted based on two *coupled* dynamic free energies for penetrant and matrix, respectively (see **Figure 1c** of the main text). In the dilute penetrant limit of interest here, the polymer matrix dynamics is described by its own dynamic free energy calculated based on bulk ECNLE theory[32-34] as recently extended to crosslinked polymer networks[5]. A tangent Koyama[35, 36] semiflexible chain model is adopted where polymer beads interact with beads on other polymers and with a spherical penetrant via a site-site hard-core repulsion (**Figure 1c** of the main text). The structural correlations that enter the dynamical theories are computed using the polymer reference interaction site model (PRISM) integral equation theory[5, 28, 34].

Per prior work[5], this athermal model is related to real thermal polymer networks by employing a well-known mapping procedure that equates the dimensionless compressibility computed from PRISM theory to its temperature dependent experimental analog obtained from PnBA equation-of-state data at 1 atm[5, 37]. This produces an a priori relation between packing fraction and absolute temperature. Crosslinking is modeled dynamically as immobile (pinned strictly or vibrate locally) bare beads of the Koyama semiflexible chain distributed in a regular manner along a chain per the schematic in **Figure 1c** of the main text. Pinning is taken to not modify the structural correlations relative to the baseline polymer melt, consistent with recent simulation findings[38]. Prior quantitative comparisons of the predictions of ECNLE theory for the alpha time of Kuhn segments (deduced from a Kramers mean first passage time calculation[39, 40] including collective elastic effects[5, 34]) in a PnBA crosslink network show excellent agreement with experiment and simulation[5]. This provides the foundational input required in SCCH theory[28-31] to construct the penetrant dynamic free energy which is coupled with the matrix dynamic free energy (see **Figure 1c** of the main text).

All technical details and equations concerning the implementation of SCCH theory can be found in the literature[28-31]. Briefly, the penetrant alpha process is of a coupled local-nonlocal spatial character, involving hopping over a local cage barrier ($F_{B,p}$) due to its local interactions due to neighboring polymer matrix particles (Kuhn segments), which is coupled with small, but long range, collective displacements of all matrix particles outside the cage characterized by an elastic barrier ($F_{el,p}$). When the temperature is relatively high (weakly supercooled regime) or the penetrant-to-matrix size ratio is small, the elastic barrier is negligible, and the local cage barrier dominates the penetrant hopping or alpha relaxation process. But in the deeply supercooled regime for sufficiently large penetrants, both the local cage and elastic barrier are crucial. The theory self-consistently predicts the magnitude of facilitating activated dynamic fluctuations of the matrix that are required to achieve a penetrant hop over its barrier, yielding a dependence of the penetrant activated relaxation time on the crosslink density, penetrant-to-matrix size ratio, and temperature. SCCH theory focuses on the local penetrant hopping event and does not explicitly address geometric mesh confinement effects on penetrant diffusivity.

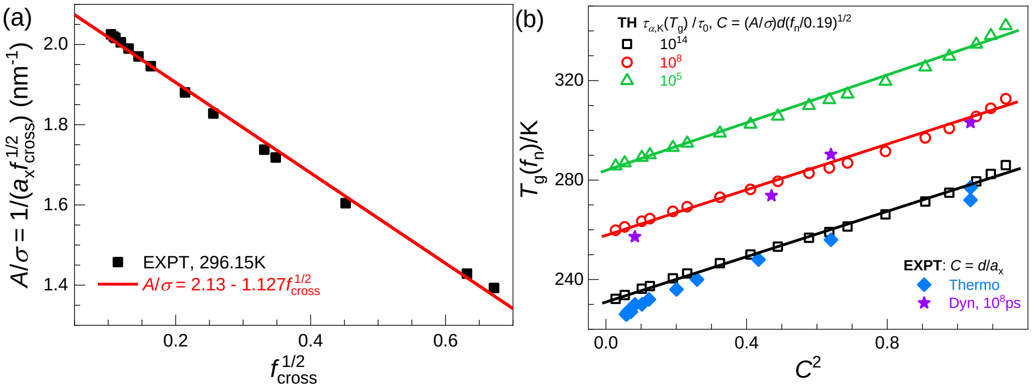

Figure S5. (a) The prefactor $A/\sigma = 1/(a_x f_{\text{cross}}^{1/2})$ in units of inverse nm for PnBA networks as a function of the square root of crosslink density $f_{\text{cross}} \equiv f_{\text{n}}/0.19$. The red line is a linear fit, $A/\sigma = 2.13 - 1.127(f_{\text{n}}/0.19)^{1/2}$, that is used as input in TH to calculate the confinement parameter as $C = (A/\sigma)d(f_{\text{n}}/0.19)^{1/2}$ and the corresponding penetrant diffusion constant. Because f_{cross} and a_x are negligibly affected by temperature, the fitting formula has no temperature dependence. (b) Glass transition temperatures defined based on different timescale criteria in TH and EXPT as a

function of squared confinement parameter, C^2. Note, C is computed based on $C = (A/\sigma)d(f_{\mathrm{n}}/0.19)^{1/2}$ and $C = d/a_x$ in the TH and EXPT, respectively.

In experiments and simulations, one can define the confinement parameter as $C = d/a_{\mathrm{x}}$ and compute the mesh size directly, as discussed previously[23, 41]. However, this approach is not directly applicable in the theory since our modeling of crosslinks via the neutral pinning of beads along the polymer chain does not directly allow the evaluation of a_{x}. In experiments, the mesh size a_{x} is assumed proportional to the square root of the mean number of bare beads between two neighboring crosslinks N_{x}, i.e., $a_{\mathrm{x}} \propto \sigma N_{\mathrm{x}}^{1/2}$. Based on our recent study of PnBA crosslinked networks[5], the experimental crosslink fraction was defined as $f_{\mathrm{cross}} = 1/N_{\mathrm{x}}$ which is used in the present work. As such, one obtains $a_{\mathrm{x}} \propto \sigma/f_{\mathrm{cross}}^{1/2}$, and hence we take the confinement parameter to be $C = d/a_{\mathrm{x}} = A(d/\sigma)f_{\mathrm{cross}}^{1/2}$, where the prefactor A is the proportionality factor between a_{x} and $\sigma/f_{\mathrm{cross}}^{1/2}$. Thus, we obtain $A/\sigma = 1/(a_{\mathrm{x}}f_{\mathrm{cross}}^{1/2})$. Based on purely experimental results for a_{x} and f_{cross}, we compute A/σ and plot it as a function of the square root of crosslink fraction $f_{\mathrm{cross}}^{1/2}$ in **Figure S5a**. Previous simulations for semi-dilute crosslinked polymer solutions[23] also adopted N_{x} to evaluate the mesh size and introduced a constant prefactor of 1.94 in defining the confinement parameter, which is the analog of the A parameter. Using the estimate of a bare bead size for PnBA as 1 nm, one obtains $A/\sigma = 1.94\ \mathrm{nm}^{-1}$. In **Figure S5a**, we find the change of A/σ is very limited, decreasing from 2.0 to 1.4 in unit of nm^{-1}, consistent in magnitude with $1.94\ \mathrm{nm}^{-1}$ but our A/σ does have a weak crosslink fraction dependence. An apparent linear behavior is observed between A/σ and $f_{\mathrm{cross}}^{1/2}$ (see red line in **Figure S5a**), given as $A/\sigma\ (\mathrm{nm}^{-1}) = 2.13 - 1.127 f_{\mathrm{cross}}^{1/2}$. Using this $A/\sigma\ (\mathrm{nm}^{-1})$ as input, we define the confinement parameter in our theoretical analysis as $C = [2.13 - 1.127(f_{\mathrm{n}}/0.19)^{1/2}]d(f_{\mathrm{n}}/0.19)^{1/2}$, where the penetrant diameter d for real systems is given in **Table 1** of the main text. Importantly, we know that

$f_{\rm cross} = f_{\rm n}/0.19$ from our previous quantitative study of pure PnBA melts and crosslinked networks[5], and empirically find that the crosslink density $f_{\rm n}$ in our theory is proportional to $f_{\rm cross}$ in experiment with a multiplier of 0.19.

II. Role of Segmental Relaxation: $D_{\rm p}$ versus $T_{\rm g}(f_{\rm n})$

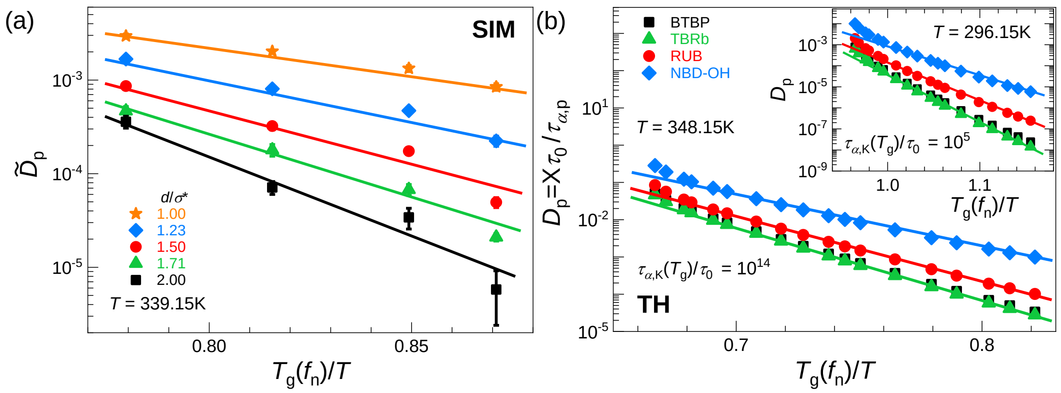

Figure S6. Relationship between penetrant diffusivity and $T_{\rm g}(f_{\rm n})$ at higher temperatures than studied in EXPT. Diffusion constants for the different size penetrants are plotted as a function of $T_{\rm g}(f_{\rm n})/T$ over a wide range of crosslink fractions for temperatures different than **Figure 2** in the main text for (a) SIM at $T = 339.15$K and (b) TH at $T = 348.15$K, with $D_{\rm p} = X(\tau_0/\tau_{\alpha,\rm p})$ in the TH and $X \equiv \frac{\exp(-C^2)}{C}$. In the inset of (b), we also show $D_{\rm p}$ at $T = 296.15$K as a function of $T_{\rm g}(f_{\rm n})/T$ based on different $T_{\rm g}$ criteria: $\tau_{\alpha,\rm K}(T_{\rm g})/\tau_0 = 10^5$ (essentially identical to that used in the simulations) which is different from that in the main frame where $\tau_{\alpha,\rm K}(T_{\rm g})/\tau_0 = 10^{14}$ is adopted in the spirit of the typical experimental vitrification definition that the polymer alpha relaxation time equals 100 s.

Taking the diffusion constant as a product of the inverse alpha time $\tau_0/\tau_{\alpha,\rm p}$ and the direct entropic mesh confinement contribution formula $X = \exp(-C^2)/C$, i.e., eq (1) of the main text with b = 1, we can theoretically calculate the probe diffusivity for the four organic dyes as shown in **Figure 2c** of the main text (solid symbols). One finds the combination of activated segmental relaxation physics (i.e., the penetrant hopping time contribution originates from the coupling of its motion with the segmental relaxation process) and entropic mesh confinement effects does not modify the exponential form of the relationship (penetrant diffusion constant or inverse alpha time

versus T_g/T), although the corresponding apparent activation energy quantitatively increases (see **Figure 2d** of the main text) upon including the mesh confinement contribution as it must. When varying the temperature, the entropic confinement parameter contribution remains unchanged, while the activated hopping contribution changes a lot. This is shown in **Figure S6b** for the theoretical predictions at a higher temperature where one sees that the exponential form of the relationship remains unchanged, but the slope decreases with temperature, consistent with the simulation findings in **Figure 2b** of the main text and **Figure S6a**.

Finally, **Figures S7a and S7b** presents a plot of the apparent dimensionless slope, k_{cross}, versus the penetrant-to-matrix size ratio, d/σ, from **Figure 2d** in both log-log and linear-linear formats, respectively. One sees both plots exhibit rather good linearity. This suggests an alternative linear behavior that differs from the logarithmic relationship in **Figure 2d** is also reasonable, although it is accurate over a more limited range of the size ratio parameter, d/σ. Moreover, by combining the good linear relationship between k_{cross} and d/σ in **Figures S7b** with our finding that $\log(1/D_{\mathrm{p}}) \propto T_{\mathrm{g}}(f_{\mathrm{cross}})$ in the main text, a linear relationship between $\log(D_{\mathrm{p}})$ and d/σ can be deduced which is distinct from the power law dependence discussed in the main text (**Figure 3**). As predicted by SCCH theory and tested in experiments for molecular liquids or polymer melts[37], this linear relation only holds over limited range of relatively small values of d/σ. In contrast, the power law relationship works well over a wider range of d/σ, as confirmed here in the presence of strong crosslinking (see **Figures 3a** and **3b** of the main text and **Figures S10c** and **S10d** for the power law relation, and **Figures S9a**, **S9b**, **S10a**, and **S10b** for the linear relation).

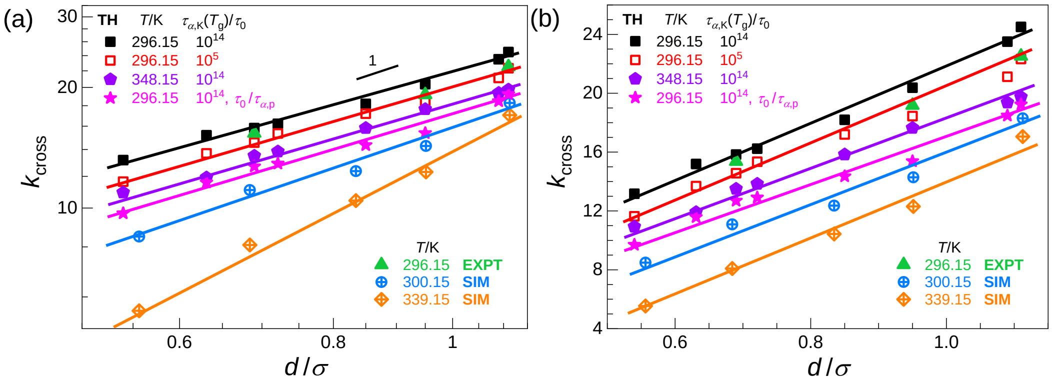

Figure S7. Apparent dimensionless slopes k_{cross} of $\log(1/D_{\mathrm{p}})$ versus $T_{\mathrm{g}}(f_{\mathrm{n}})/T$, as a function of size ratio d/σ plotted in a (a) log-log and (b) linear-linear manners for different temperatures and methods (EXPT, SIM, TH). For the theoretical calculation, we also present the corresponding slope deduced from a $\log(\tau_{\alpha,\mathrm{p}}/\tau_0)$ versus $T_{\mathrm{g}}(f_{\mathrm{n}})/T$ plot. Note: as in **Figure 2d** of the main text, the x-axes of SIM data is multiplied by the factor 0.573/1.03.

III. Penetrant-to-Matrix Size Ratio Dependence

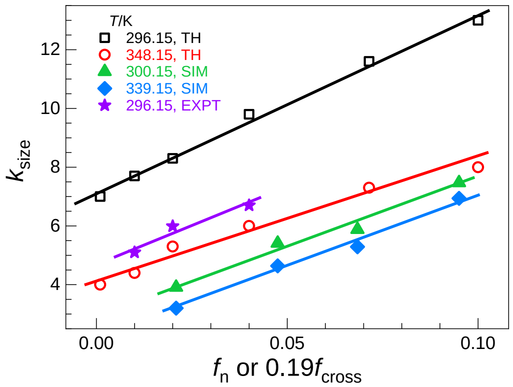

Figure S8. Linear-linear plot of the crosslink density dependence of the power law exponent for the D_{p} versus d/σ plot, i.e., the slope of $\log(1/D_{\mathrm{p}})$ versus $\log(d/\sigma)$, in EXPT, TH and SIM at various temperatures.

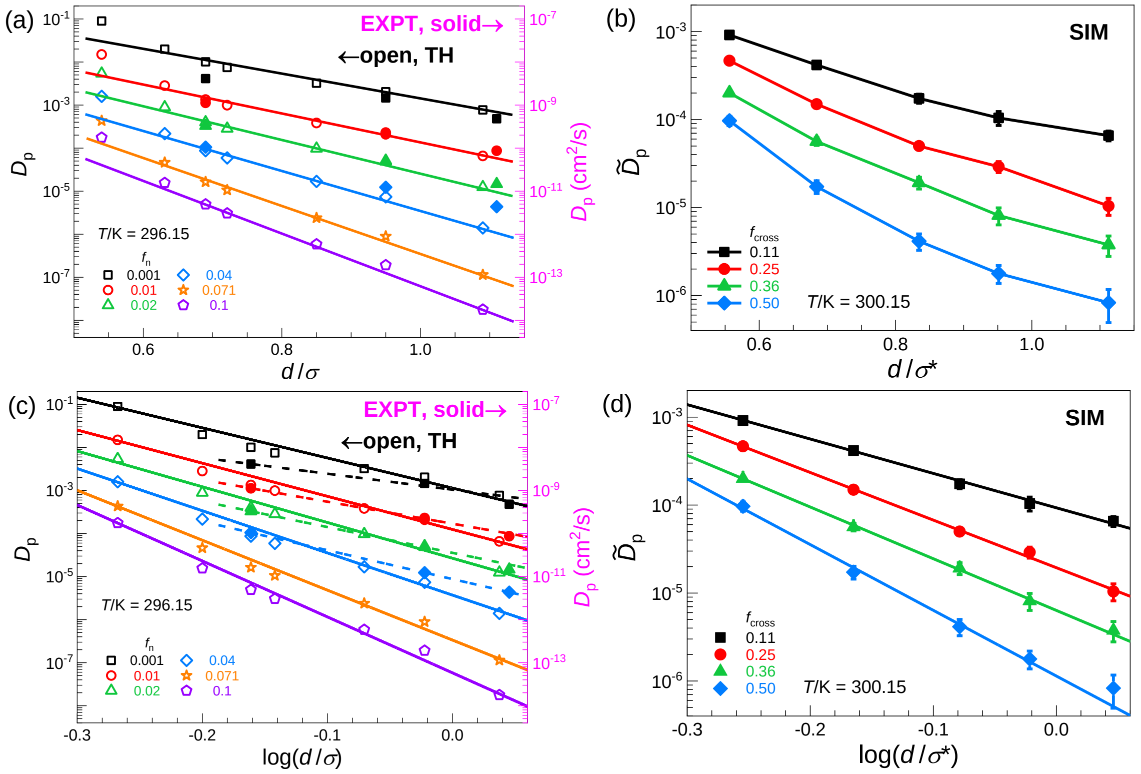

Figure S9. Penetrant size ratio dependence of its diffusivity plotted in log-linear manner at various fixed crosslink densities as a function of size ratio d/σ in (a) TH at $T = 296.15$K and (b) SIM at $T = 300.15$ K. The simulation value of d/σ is multiplied by the factor of 0.573/1.03 for comparison with the TH and EXPT results. (c) and (d) are the same displays as in (a) and (b), respectively, but as a function of $\log(d/\sigma)$.

For the model of nanoparticle diffusion in rubbery polymer networks[23, 41, 42], when the confinement parameter is small enough there is no entropic barrier, and a power law relation between spherical particle diffusion constant and its diameter is predicted based on a hydrodynamic argument (Stokes relation). There are three differences between our SCCH theory and this approach: (i) Our prediction (and also our simulation and experimental results) applies solely to larger size penetrants; (ii) The physical relaxation mechanism underlying our power law relationship originates from penetrant activated hopping dynamics, not hydrodynamics in a non-activated regime where the confinement parameter is very low; (iii) The power law exponent for the model of Ref.41 is 3, generally much smaller than our crosslink density dependent exponent due to the qualitatively different physics (see **Figure S8**).

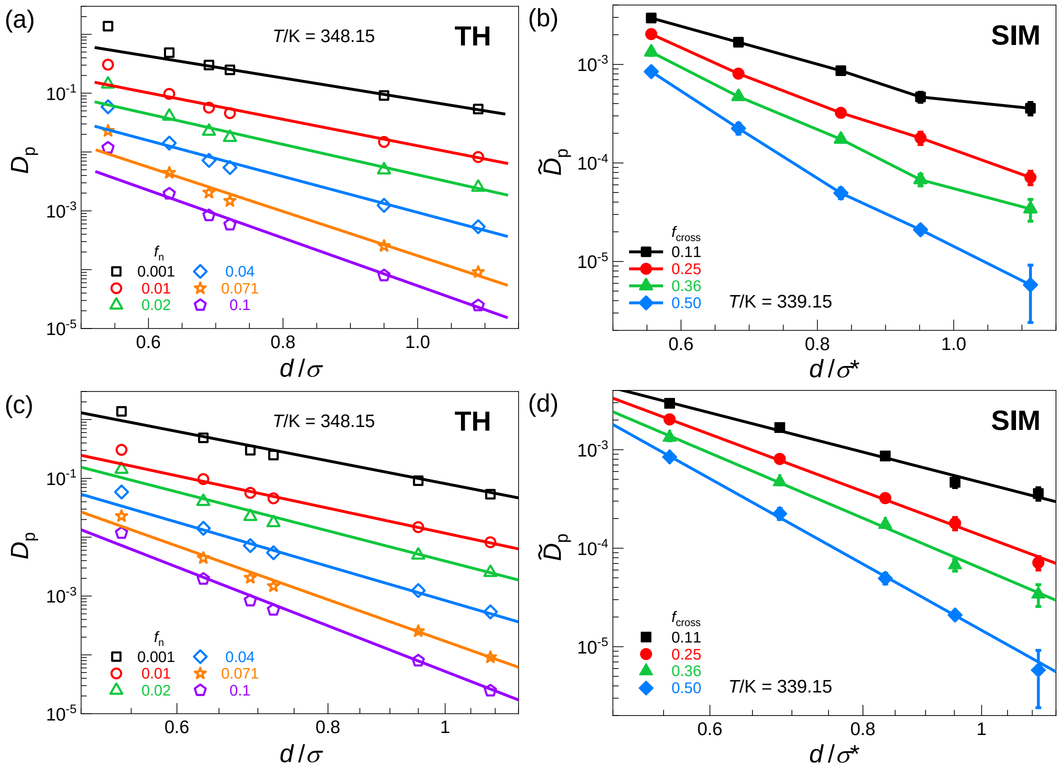

Figure S10. Size ratio dependence of the penetrant diffusion constant at a higher temperature than studied experimentally. (a) and (b) are the same displays as **Figures S9a** and **S9b**, respectively, but for an elevated temperature (note that EXPT data for a higher temperature is not included): (a) TH at $T = 348.15\,\mathrm{K}$ and (b) SIM at $T = 339.15\,\mathrm{K}$. The simulation value of d/σ is again multiplied by the factor 0.573/1.03 for comparison with TH and EXPT. (c) and (d) are the same displays as (a) and (b), respectively, but in a log-log manner.

Although the exponential relationship between diffusion constant and penetrant-to-matrix size ratio in the smaller $d/\sigma < 0.5$ regime has been previously discussed and experimentally confirmed for chemical diverse penetrants in various polymer melts[37] (and in simulation[43]), to date the power law relationship lacks direct experimental and/or simulation evidence under variable crosslink density conditions. In the main text we provided a first simulation test of the power law relationship (since only the large $d/\sigma > 0.5$ regime is probed in our present work) between penetrant diffusivity and its size in crosslinked networks. The simulations find that for each crosslink density at a medium supercooled temperature ($T = 300.15$K) the predicted power law relationship is well obeyed (see **Figure 3b**). Moreover, the absolute value of the slope in the log-log plot (the power

law exponent) increases linearly with crosslink density as shown in **Figure S8** where log $(1/D_{\mathrm{p}}) \propto T_{\mathrm{g}}(f_{\mathrm{cross}})$, $C^2 \propto T_{\mathrm{g}}(f_{\mathrm{n}})$ and $C = A(d/\sigma)f_{\mathrm{cross}}^{1/2}$ are combined and the very weak dependence of the prefactor A on crosslink density is ignored (see **Figure S5a**, A/σ only varies 30%, from 2.0 to 1.4 in unit of nm^{-1}, while $f_{\mathrm{cross}}^{1/2}$ changes by a factor of ~7). This good agreement between simulation and theory provides additional support for the physical picture predicted by SCCH theory.

The question of an alternative linear relationship between log (D_{p}) and d/σ is addressed in **Figure S9** for both the theory and simulation. It is found to be valid only over a limited range of low d/σ ratios. Finally, the effect of temperature is considered in **Figure S10**, and our conclusions drawn at a single experimental temperature are found to remain robust.

IV. Confinement Parameter Dependence

Figure 4c of the main text presented SCCH theory predictions in the plotting format $CD_{\mathrm{p}}\sim \exp(-BC^2)$, which was shown to work rather well not only in the low C regime, but also in the higher C regime. This is nontrivial since in the low C regime only local caging of a penetrant by the surrounding polymer segments is present, while in the larger C regime both local caging and the facilitation effect of longer-range elastic cooperative motion of Kuhn segments is important. However, in *both* cases SCCH theory predicts the same C −dependence of the penetrant diffusion constant. This seems consistent with our experimental and simulation results in **Figures 4a** and **4b**, respectively, that find an Arrhenius-like dependence of the penetrant diffusion constant which we argue is because only the local cage barrier is significant (elastic barrier relatively small or negligible) for the present systems of interest, as seen in **Figure 5** and **Figure S14**.

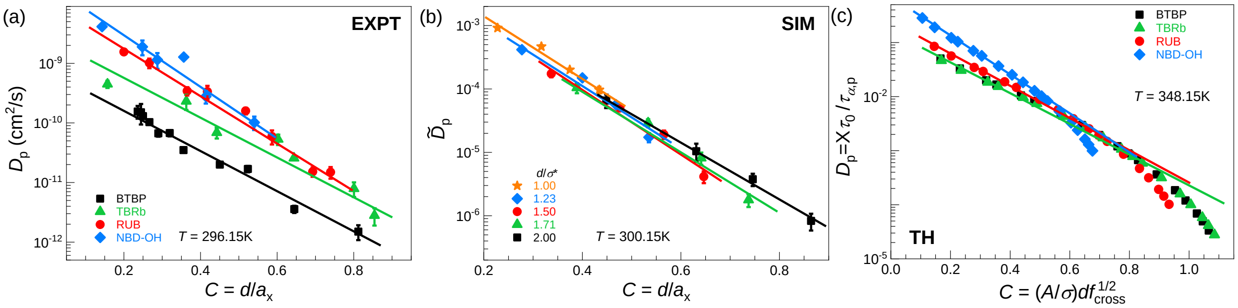

Figure S11. Log-linear plot of the penetrant diffusivity of various penetrants as a function of C in (a) EXPT, (b) SIM, and (c) TH over a wide range of crosslink fractions at a fixed medium temperature ($T = 296.15\text{K}$ for EXPT and TH, $T = 300.15\text{K}$ for SIM). Here, $C = d/a_x$ in EXPT and SIM, and $C = (A/\sigma)d(f_{\text{n}}/0.19)^{1/2}$ in TH. The simulation confinement parameter C is multiplied by the factor 0.573/1.0 for comparison with EXPT and TH.

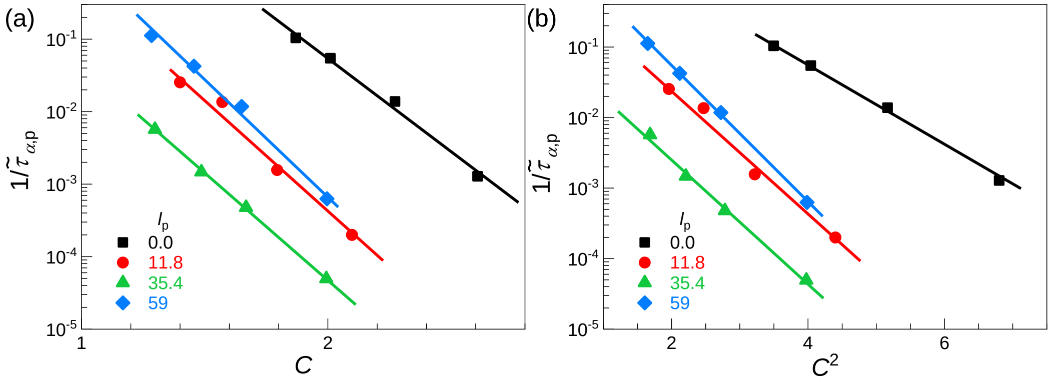

Figure S12. Literature simulation data[42] for the inverse penetrant alpha time as a function of the confinement parameter, plotted versus (a) C and (b) C^2 for a crosslinked network of semiflexible polymers of very different persistent lengths l_{p} over a range of crosslink densities at a fixed penetrant size ($d = 6.4r_{\text{c}}$ with r_{c} being the simulation length unit).

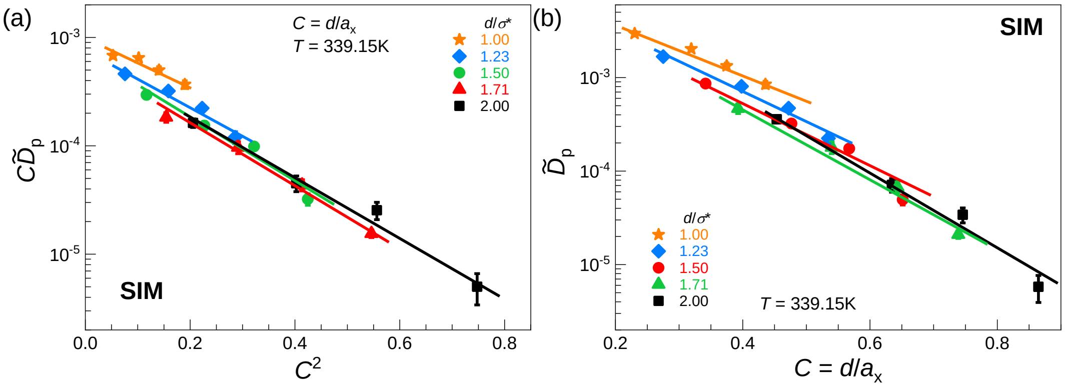

Figure S13. Relationship between penetrant diffusivity and confinement parameters at higher temperature. (a) and (b) SIM data in the same display format as **Figure 4** of the main text and **Figure S11b** adopted to test the relationships $CD_{\text{p}} \sim \exp(-BC^2)$ and $D_{\text{p}} \sim \exp(-EC)$, respectively, at a higher temperature $T = 339.15\text{K}$.

Figures S11a and **S11b** present the corresponding results of D_p for experiment and simulation, respectively, for various penetrants as a function of the confinement parameter C at an intermediate temperature. An exponential relationship works reasonably well in the limited data range of $0.15 < C < 0.9$, and essentially just as well as the relationship $CD_\mathrm{p} \sim \exp(-BC^2)$, signaling another "degeneracy of interpretation". The corresponding SCCH theory result is shown in **Figure S11c**, and in the same limited C range an exponential relationship between of D_p and C is predicted by the theory. However, the exponential relation breaks down (predicted curve bends down) beyond a sufficiently large confinement parameter. To understand this behavior, we plot the penetrant local cage and elastic barriers as a function of confinement parameter C in **Figure S14b**. One sees that when C is not too large the elastic barrier is negligible and penetrant dynamics is determined by local cage barrier which scales linearly with C. Thus, the $D_\mathrm{p} \sim \exp(-EC)$ behavior is predicted to apply in the weakly or intermediately supercooled regime and/or for small size penetrants with E a constant prefactor.

Finally, we find that all the above observations and predictions remain robust when changing temperature, as shown in **Figures S13a** and **S13b** for the simulation data which tests the relationships $CD_\mathrm{p} \sim \exp(-BC^2)$ and $D_\mathrm{p} \sim \exp(-EC)$, respectively, at a higher temperature. However, at a higher temperature, the activated hopping contribution to penetrant diffusivity does make a quantitatively smaller contribution relative to the entropic mesh confinement effects, as physically expected. This results in the slope B discussed in the main text changing relatively weakly if the penetrant size is varied, as observed in our experiments, simulations, and theory calculations.

V. Theoretical Barriers and Physical Mechanisms

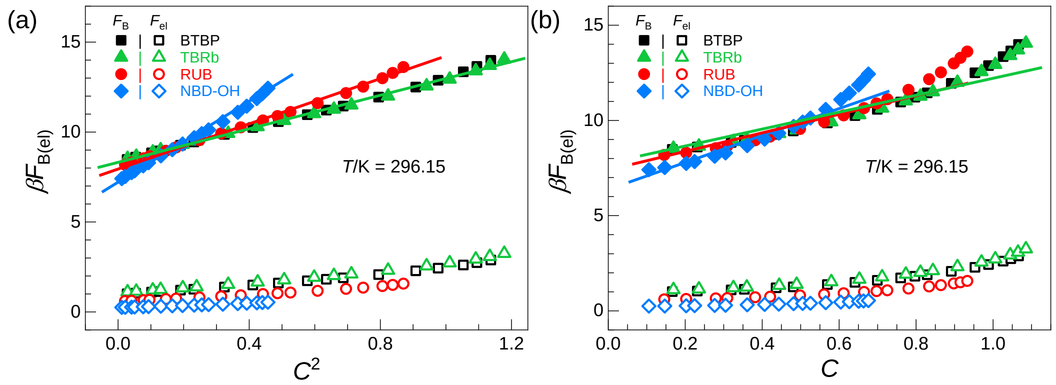

Figure S14. Theoretical predictions for the activation barriers in units of thermal energy. (a) Local cage and elastic barriers for the four molecular penetrants plotted as a function of C^2 with $C = (A/\sigma)d(f_n/0.19)^{1/2}$ over a wide range of crosslink fractions at a fixed medium temperature $T = 296.15$K. (b) Same display as in (a) but with C^2 replaced by confinement parameter C.

For all four molecular penetrants studied experimentally, their respective elastic barriers are predicted to be far smaller than their respective local cage barriers. There are several reasons for this: (i) compared to hard sphere fluids, for polymer systems the elastic barrier is relatively smaller because of the influence of chain connectivity as previously discussed[44]; (ii) the penetrant sizes (listed in **Table 1**) are relatively small compared to the Kuhn segment length of 1.72 nm, which is predicted in polymer melts[28] to result in rather strong decoupling between the penetrant and matrix dynamics and thus a low value of the elastic barrier; and (iii) crosslinking decreases the relative importance of elastic barrier compared to the local cage barrier. These effects collectively underlie the trends in **Figure 5a** of the main text and **Figure S14** where the elastic barrier increases much *slower* with $T_g(f_n)/T$, C^2 or C relative to that of local cage barrier.

References.

(1) Sheridan, G. S.; Evans, C. M. Understanding the Roles of Mesh Size, T_g, and Segmental Dynamics on Probe Diffusion in Dense Polymer Networks. *Macromolecules* **2021,** *54*, 11198-11208.

(2) Fery-Forgues, S.; Fayet, J.-P.; Lopez, A. Drastic Changes in the Fluorescence Properties of NBD Probes with the Polarity of the Medium: Involvement of a TICT State? *J. Photochem. Photobiol. A* **1993,** *70*, 229-243.

(3) Bondi, A. van der Waals Volumes and Radii. *J. Phys. Chem.* **1964,** *68*, 441-451.

(4) Zhao, Y. H.; Abraham, M. H.; Zissimos, A. M. Fast Calculation of van der Waals Volume as a Sum of Atomic and Bond Contributions and Its Application to Drug Compounds. *J. Org. Chem.* **2003,** *68*, 7368-7373.

(5) Mei, B.; Lin, T.-W.; Sheridan, G. S.; Evans, C. M.; Sing, C. E.; Schweizer, K. S. Structural Relaxation and Vitrification in Dense Crosslinked Polymer Networks: Simulation, Theory and Experiment. *Macromolecules* **2022,** *55*, 4159−4173.

(6) Grest, G. S. Communication: Polymer Entanglement Dynamics: Role of Attractive Interactions. *J. Chem. Phys.* **2016,** *145*, 141101.

(7) Plaza-Rivera, C. O.; Nguyen, H. T.; Hoy, R. S. Isostaticity and the Solidification of Semiflexible Polymer Melts. *Soft Matter* **2017,** *13*, 7948-7952.

(8) Vargas-Lara, F.; Douglas, J. F. Fiber Network Formation in Semi-Flexible Polymer Solutions: An Exploratory Computational Study. *Gels* **2018,** *4*, 27.

(9) Milchev, A.; Nikoubashman, A.; Binder, K. The Smectic Phase in Semiflexible Polymer Materials: A Large Scale Molecular Dynamics Study. *Comp. Mater. Sci.* **2019,** *166*, 230-239.

(10) Huang, J.-H.; Sun, D.-D.; Lu, R.-X. Glass Transition and Dynamics of Semiflexible Polymer Brushes. *Phys. Chem. Chem. Phys.* **2021,** *23*, 13895-13904.

(11) Zhang, T.; Riggleman, R. A. Thickness-Dependent Mechanical Failure in Thin Films of Glassy Polymer Bidisperse Blends. *Macromolecules* **2021,** *55*, 201-209.

(12) Jain, T. S.; de Pablo, J. J. Role of Local Structure on Motions on the Potential Energy Landscape for a Model Supercooled Polymer. *J. Chem. Phys.* **2005,** *122*, 174515.

(13) Riggleman, R. A.; Schweizer, K. S.; Pablo, J. J. d. Nonlinear Creep in a Polymer Glass. *Macromolecules* **2008,** *41*, 4969-4977.

(14) Simmons, D. S.; Douglas, J. F. Nature and Interrelations of Fast Dynamic Properties in a Coarse-Grained Glass-Forming Polymer Melt. *Soft Matter* **2011,** *7*, 11010-11020.

(15) Ye, Z.; Riggleman, R. A. Molecular View of Cavitation in Model-Solvated Polymer Networks. *Macromolecules* **2020,** *53*, 7825-7834.

(16) Minina, E.; Sánchez, P.; Likos, C.; Kantorovich, S. The Influence of the Magnetic Filler Concentration on the Properties of a Microgel Particle: Zero-Field Case. *J. Magn. Magn. Mater.* **2018,** *459*, 226-230.

(17) Moreno, A. J.; Verso, F. L. Computational Investigation of Microgels: Synthesis and Effect of the Microstructure on the Deswelling Behavior. *Soft Matter* **2018,** *14*, 7083-7096.

(18) Bennemann, C.; Paul, W.; Baschnagel, J.; Binder, K. Investigating the Influence of Different Thermodynamic Paths on the Structural Relaxation in a Glass-Forming Polymer Melt. *J. Phys. Condens. Mater.* **1999,** *11*, 2179.

(19) Riggleman, R. A.; Lee, H.-N.; Ediger, M. D.; De Pablo, J. J. Free Volume and Finite-Size Effects in a Polymer Glass under Stress. *Phys. Rev. Lett.* **2007,** *99*, 215501.

(20) Shavit, A.; Riggleman, R. A. Influence of Backbone Rigidity on Nanoscale Confinement Effects in Model Glass-Forming Polymers. *Macromolecules* **2013,** *46*, 5044-5052.

(21) Diaz Vela, D.; Simmons, D. S. The Microscopic Origins of Stretched Exponential Relaxation in Two Model Glass-Forming Liquids as Probed by Simulations in the Isoconfigurational Ensemble. *J. Chem. Phys.* **2020,** *153*, 234503.

(22) Mangalara, J. H.; Mackura, M. E.; Marvin, M. D.; Simmons, D. S. The Relationship between Dynamic and Pseudo-Thermodynamic Measures of the Glass Transition Temperature in Nanostructured Materials. *J. Chem. Phys.* **2017,** *146*, 203316.

(23) Sorichetti, V.; Hugouvieux, V.; Kob, W. Dynamics of Nanoparticles in Polydisperse Polymer Networks: From Free Diffusion to Hopping. *Macromolecules* **2021,** *54*, 8575-8589.

(24) Plimpton, S. Fast Parallel Algorithms for Short-Range Molecular Dynamics. *J. Comput. Phys.* **1995,** *117*, 1-19.

(25) Nosé, S. A Unified Formulation of the Constant Temperature Molecular Dynamics Methods. *J. Chem. Phys.* **1984,** *81*, 511-519.

(26) Hoover, W. G. Canonical Dynamics: Equilibrium Phase-Space Distributions. *Phys. Rev. A* **1985,** *31*, 1695.

(27) Nosé, S. An Extension of the Canonical Ensemble Molecular Dynamics Method. *Mol. Phys.* **1986,** *57*, 187-191.

(28) Mei, B.; Schweizer, K. S. Theory of the Effects of Specific Attractions and Chain Connectivity on the Activated Dynamics and Selective Transport of Penetrants in Polymer Melts. *Macromolecules* **2022,** *55*, 9134–9151.

(29) Zhang, R.; Schweizer, K. S. Correlated Matrix-Fluctuation-Mediated Activated Transport of Dilute Penetrants in Glass-Forming Liquids and Suspensions. *J. Chem. Phys.* **2017,** *146*, 194906.

(30) Mei, B.; Schweizer, K. S. Activated Penetrant Dynamics in Glass Forming Liquids: Size Effects, Decoupling, Slaving, Collective Elasticity and Correlation with Matrix Compressibility. *Soft Matter* **2021,** *17*, 2624-2639.

(31) Mei, B.; Schweizer, K. S. Theory of the Effect of External Stress on the Activated Dynamics and Transport of Dilute Penetrants in Supercooled Liquids and Glasses. *J. Chem. Phys.* **2021,** *155*, 054505.

(32) Mirigian, S.; Schweizer, K. S. Elastically Cooperative Activated Barrier Hopping Theory of Relaxation in Viscous Fluids. I. General Formulation and Application to Hard Sphere Fluids. *J. Chem. Phys.* **2014,** *140*, 194506.

(33) Mei, B.; Zhou, Y.; Schweizer, K. S. Thermodynamics–Structure–Dynamics Correlations and Nonuniversal Effects in the Elastically Collective Activated Hopping Theory of Glass-Forming Liquids. *J. Phys. Chem. B* **2020,** *124*, 6121-6131.

(34) Zhou, Y.; Mei, B.; Schweizer, K. S. Activated Relaxation in Supercooled Monodisperse Atomic and Polymeric WCA Fluids: Simulation and ECNLE Theory. *J. Chem. Phys.* **2022,** *156*, 114901.

(35) Koyama, R. Light Scattering of Stiff Chain Polymers. *J. Phys. Soc. Jpn.* **1973,** *34*, 1029-1038.

(36) Honnell, K. G.; Curro, J. G.; Schweizer, K. S. Local Structure of Semiflexible Polymer Melts. *Macromolecules* **1990,** *23*, 3496-3505.

(37) Mei, B.; Sheridan, G. S.; Evans, C. M.; Schweizer, K. S. Elucidation of the Physical Factors That Control Activated Transport of Penetrants in Chemically Complex Glass-Forming Liquids. *Proc. Natl. Acad. Sci. USA* **2022,** *119*, e2210094119.

(38) Zheng, X.; Guo, Y.; Douglas, J. F.; Xia, W. Understanding the Role of Cross-Link Density in the Segmental Dynamics and Elastic Properties of Cross-Linked Thermosets. *J. Chem. Phys.* **2022,** *157*, 064901.

(39) Kramers, H. A. Brownian Motion in a Field of Force and the Diffusion Model of Chemical Reactions. *Physica* **1940,** *7*, 284-304.

(40) Hänggi, P.; Talkner, P.; Borkovec, M. Reaction-Rate Theory: Fifty Years after Kramers. *Rev. Mod. Phys.* **1990,** *62*, 251-341.

(41) Cai, L.-H.; Panyukov, S.; Rubinstein, M. Hopping Diffusion of Nanoparticles in Polymer Matrices. *Macromolecules* **2015,** *48*, 847-862.

(42) Xu, Z.; Dai, X.; Bu, X.; Yang, Y.; Zhang, X.; Man, X.; Zhang, X.; Doi, M.; Yan, L.-T. Enhanced Heterogeneous Diffusion of Nanoparticles in Semiflexible Networks. *ACS nano* **2021,** *15*, 4608-4616.

(43) Kanduč, M.; Kim, W. K.; Roa, R.; Dzubiella, J. How the Shape and Chemistry of Molecular Penetrants Control Responsive Hydrogel Permeability. *ACS nano* **2020,** *15*, 614-624.

(44) Mei, B.; Zhou, Y.; Schweizer, K. S. Experimental Tests of a Theoretically Predicted Noncausal Correlation between Dynamics and Thermodynamics in Glass-Forming Polymer Melts. *Macromolecules* **2021,** *54*, 10086-10099.
